# Decision‐Making in Public Health Crises: A Qualitative Systematic Review and Meta‐Ethnography

**DOI:** 10.1002/puh2.70358

**Published:** 2026-09-15

**Authors:** Ehmaidy Al qaf'an, Stewart Alford, Holly A. Mack, David Lim

**Affiliations:** ^1^ Improving Palliative, Aged and Chronic Care Through Clinical Research and Translation University of Technology Sydney Ultimo New South Wales Australia; ^2^ Kaplan Business School Kaplan Australia Brisbane Queensland Australia; ^3^ Translational Health Research Institute Western Sydney University Campbelltown New South Wales Australia

**Keywords:** COVID‐19, decision analysis, decision‐making, disaster management, health leaders, pandemic, public health crisis

## Abstract

**Introduction:**

Decision‐making (DM) during public health crises occurs under uncertainty, time pressure and resource constraints. Although numerous studies examine leadership and governance during crises, evidence remains fragmented and has not been systematically synthesised to integrate the DM models used in practice with the factors that shape those decisions. This qualitative systematic review addresses this gap by examining how health system leaders make decisions during public health crises.

**Methods:**

A qualitative systematic review using a meta‐ethnographic approach was conducted in accordance with Preferred Reporting Items for Systematic Reviews and Meta‐Analyses (PRISMA) 2020 guidelines. The protocol was registered in PROSPERO (CRD42023475382). PubMed, Medline, Embase, Scopus, Business Source, CINAHL and ProQuest were searched for English‐language qualitative studies published since 1962.

**Results:**

Twenty‐one studies were included with seven DM models and eight prioritised decision factors identified. Adaptive and evidence‐based models predominated, reflecting the need to act under conditions of urgency while continuously incorporating emerging information. Knowledge and urgency emerged as key, interdependent drivers shaping model selection, highlighting that effective crisis DM depends not only on the choice of model but also on how the tension between rapid action and informed judgement is managed.

**Limitations:**

Non‐English literature was excluded, and the evidence base was dominated by COVID‐19 studies and developed‐country settings, which may limit transferability to lower resource contexts.

**Conclusion:**

This review provides an integrated synthesis of DM models and prioritised decision factors during public health crises. The findings underscore the importance of strengthening real‐time evidence systems and adaptive decision capacity to support timely, accountable and context‐sensitive crisis decisions.

## Graphical abstract

This graphical abstract highlights seven decision‐making models and eight prioritised factors shaping public health crisis decisions. Knowledge and urgency emerged as central drivers, underscoring the need for adaptive, evidence‐informed, ethical and context‐sensitive decision systems.

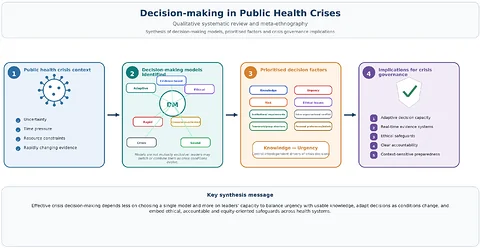

## 1 Introduction

In the event of a health crisis, timely and effective decision‐making (DM) is critical for mitigating threats, saving lives and managing resources efficiently [1]. Yet decisions made during a crisis are rarely isolated: Actions taken in one part of the system can trigger downstream effects across service delivery, workforce capacity and population trust, highlighting the value of a systems‐thinking perspective in understanding crisis response [2, 3, 4]. A public health crisis can disrupt the fundamentals of medical care, limit or strain healthcare resources, cause inefficient service delivery and access issues and result in inadequate care [5, 6]. In‐crisis DM has attracted relatively limited attention despite the recent COVID‐19 pandemic [7]. Existing frameworks proposed to guide crisis DM in complex public health environments include systems thinking approaches that emphasise interdependence, feedback and unintended consequences in health systems [2, 3, 4]. From a crisis governance perspective, decision‐makers also face core leadership challenges such as sensemaking, coordination across organisations and levels of government and maintaining legitimacy and confidence while implementing urgent measures [8, 9]. These conditions help explain why a variety of DM models may be applied before, during and post crises.

Despite the growing body of literature on crisis DM, existing studies remain fragmented. Prior work has largely focused on specific leadership experiences, single decision frameworks or narrow crisis contexts [10, 11, 12, 13]. Moreover, the evidence remains fragmented surrounding how DM models are integrated within governance processes, leadership capabilities or organisational responses in the context of crisis, rather than explicitly identified and compared across crisis contexts [14, 15, 16, 17]. Existing reviews have primarily examined pandemic governance or synthesised leadership characteristics required during the COVID‐19 era but have not systematically consolidated the DM models used by health system leaders alongside the decision variables prioritised during public health crises in general [18, 19]. In addition, reviews of decision‐support frameworks in public health emergencies tend to focus on tools and frameworks rather than synthesising the DM models applied in practice and the contextual determinants shaping their use [20]. Similarly, systematic reviews of multi‐level governance in COVID‐19 have highlighted the breadth and fragmentation of governance studies but do not consolidate the DM models used by health system leaders or the factors prioritised within decisions or during the making of decision [21]. Consequently, there remains limited synthesis of (i) DM models applied in crisis and (ii) factors influencing DM and decisions.

An additional gap concerns equity and context. Crisis DM evidence is often dominated by high‐income countries (HICs), whereas low‐ and middle‐income countries (LMICs) may face distinctive constraints related to data infrastructure, capacity and inequitable access to countermeasures, influencing which evidence is available and how rapidly decisions can be implemented [22, 23]. Greater inclusion of LMIC perspectives is therefore necessary to improve transferability and preparedness across settings, especially as public health crises can cross geographical boundaries, and synchronising of pan‐governmental approaches may be required.

To address this evidence gap, a qualitative systematic review using meta‐ethnography was conducted to interpretively synthesise evidence on the DM models employed by health system leaders during public health crises and the factors prioritised during DM. The review questions are: What DM models do health system leaders employ during public health crises, and which factors were prioritised in those decisions? This review is timely, as it draws on past experiences to help healthcare leaders plan and prepare for future public health crises.

## 2 Methods

A qualitative systematic review and meta‐ethnography were conducted following the Preferred Reporting Items for Systematic Reviews and Meta‐Analyses (PRISMA) 2020 and eMERGe reporting guidelines [24, 25]. The protocol for this review was registered with PROSPERO (CRD42023475382) [7]. The focus on qualitative studies was deemed particularly well suited to exploring complex DM processes, tacit reasoning and contextual influences that are not readily captured through quantitative measures. Meta‐ethnography enables the translation of concepts across qualitative studies to develop higher order interpretive explanations [26, 27].

### 2.1 Eligibility Criteria

For this review, a public health crisis is defined as a rapidly evolving threat to population health that exceeds the capacity of standard public health systems and demands immediate action under conditions of uncertainty and constraint. This aligns with the definition of emergencies provided by the National Academies of Sciences, Engineering and Medicine and concords with the World Health Organization's definition of exceptional public health challenges requiring coordinated interventions [28, 29]. Qualitative and mixed‐methods studies published in English from 1962 to 2025 were included. The index year was selected to align with and extend a previously published bibliometric analysis on leadership and crisis DM [30]. Study participants were not restricted. Although systematic reviews were not included, the reference lists of related and relevant systematic reviews (including narrative, scoping, integrated, umbrella and bibliometric) were searched for potential primary studies.

### 2.2 Data Sources

Searches on MEDLINE (Ovid), PubMed (NLM), Embase (Elsevier), ProQuest, Scopus (Elsevier) and Business Source (EBSCO) were conducted in January 2024 and updated on 12 December 2025. The reference list was subsequently checked for accuracy, completeness and currency, and the cited literature was reviewed to ensure that recent and relevant evidence was appropriately represented. The search terms used for PubMed included ‘healthcare leader’, ‘healthcare executive’, ‘healthcare administrator’ and ‘clinical leader’ to denote the population of interest; ‘emergency decision‐making’, ‘crisis management’ and ‘decision‐making process’ for the concept; and ‘hospital’, ‘community’ and ‘population health’ for the context. A comprehensive list of all search terms used is available in Supporting Information File 1, whereas the full database search history is documented in Supporting Information File 2.

### 2.3 Study Screening

Results from all database searches were loaded into Covidence systematic review software (Veritas Health Innovation, Melbourne, Australia) for record management and processing. Titles, abstracts and full‐text articles were screened independently by two reviewers (E.A. and S.A.) in Covidence. Discrepancies were resolved through discussion and, if needed, by arbitration with a third reviewer (D.L.). A kappa coefficient (a statistical measure of inter‐rater reliability) of >90% was obtained from Covidence, indicating very strong agreement.

### 2.4 Quality Assessment

E.A. and S.A. evaluated the quality of the selected studies using the JBI appraisal checklists. Disagreements were resolved through discussion, and inter‐rater agreement before consensus was strong, with a kappa value of >80%. The quality appraisal is tabulated in Supporting Information File 3.

### 2.5 Data Extraction

The JBI data extraction template [31] was modified and piloted on a subset of included studies prior to full extraction. The key extracted data are summarised in Table 1, including the aims, key findings, authors’ conclusions and limitations of each included study, whereas the comprehensive extracted data are provided in Supporting Information File 4. Table 1 provides the descriptive foundation for the synthesis by showing how the primary study evidence informed the later interpretive synthesis of DM models and prioritised decision factors. Extraction was completed by E.A. and independently audited by S.A.; disagreements were resolved by consensus and involvement of D.L.

**TABLE 1:** Extracted data from included studies (aims, key findings, authors’ conclusions and limitations).

References	Aims	Key findings	Authors’ conclusions	Limitations
[32]	Explore primary care leaders’ experiences and strategies during COVID‑19	Early: rapid change and constant decisions; team purpose buffered stress Later: layered crises (fatigue/burnout) strained relationships Key strategies: presence, over‑communication, relationship‑building, adaptive decision‑making	Relational and adaptive leadership supports teams and organisational resilience during prolonged crisis and burnout	Small sample; recruitment via authors’ networks (selection bias) Limited diversity of leaders/settings; reflexivity noted
[33]	Examine organisational DM in North Carolina during COVID‑19	Decisions shaped by scientific authorities, peer decisions and prior crisis experience Decentralisation and auxiliary bodies helped manage complex inputs Underrepresentation of racial/ethnic minority communities in the sample	Decision effectiveness depended on system ‘fit’ and adaptability Alignment and collaboration across local organisations strengthened the response	Timing effects not controlled; interviews across a rapidly evolving period; recall bias Metro/White skew limits generalisability; early‑pandemic window only
[34]	Identify how non‑profit healthcare CEOs make ethical decisions and avoid ethical scandals	Themes: promoting values, fostering ethical culture and embedding ethical decisions at all levels Encouraged diverse worldviews and safe expression of ethical concerns Policies and supportive climate reduced unintended ethical consequences	CEO strategies can reduce ethical failures and strengthen integrity and social responsibility during crises	Small, single‑region CEO sample; transferability limited Only CEOs included; sector‑specific context
[35]	Understand State Health Officials’ (SHOs) DM during COVID‑19 and identify tool/training needs	No dedicated decision tool; reliance on consensus was challenging but not avoidable COVID‑19 is viewed as the most complex emergency experienced State–local interaction and political/media dynamics strongly influenced decisions	Structured, stepwise decision tools and workforce training are needed to support SHOs during public health emergencies	SHO‑only perspectives; geographic overlap limits representativeness Qualitative interpretation and researcher presence may introduce bias
[36]	Describe Bay Area shelter‑in‑place (SIP) decisions and advocate for decision intelligence in crisis management	First regional SIP orders issued amid rising cases; high uncertainty about effectiveness Decision intelligence framed trade‑offs; consequence/strategy tables supported alternatives assessment Hospitals were not overwhelmed, though preparedness gaps were acknowledged	Rapid action under uncertainty is required; decision quality should be judged by the process at the time Ongoing decision intelligence training recommended	Limited knowledge of transmission and preparedness at decision time Outcomes alone are insufficient to evaluate decision quality
[37]	Examine long‑term care (LTC) transition DM during COVID‑19 in Ontario	Seven factors shaped transition decisions (e.g., institutional priorities, resources and beliefs) Professionals often dominated decisions; pandemic increased care‑partner involvement Emotions and resources influenced crisis appraisal and response decisions’ options	Crisis reshaped transition decisions; decision supports/aids are needed for stakeholders LTC conditions drive ongoing transitions, implying policy relevance	English‑language restriction and topic complexity may omit evidence Limited equity‑group perspectives; minimal patient/family involvement in factor identification
[38]	Develop digital health dashboards using citizen data to support rapid, ethical public health DM	Dashboards convert citizen data into real‑time evidence and analytics Enable direct communication between decision‑makers and citizens for risk management and DM Support decentralisation and data sovereignty but depend on citizen uptake	Citizen‑driven dashboards can strengthen rapid responses and equity‑oriented systems integration	Digital divides affect participation; ongoing security risks Real‑world validation needed; ethics exemption may raise concerns
[39]	Identify decision‑makers’ preferences for rapid evidence summaries in health/humanitarian crises and develop a template	Referred concise bullet points/infographics with actionable messages Time pressure and access constraints shaped evidence use in the decision Social‑media‑friendly formats improved dissemination	Plain‑language rapid summaries support real‑time decisions; templates should be tailored and include critical appraisal	Non‑users are less familiar with websites; limited comparisons Internet constraints; topic categorisation difficulties; limited time to engage with longer documents
[40]	Develop an ethical tool to guide fair resource allocation and clinical ramp‑down during COVID‑19	Stepwise ethical tool developed using Accountability for Reasonableness (A4R) Uniform first‑wave ramp‑down caused inefficiency; the second wave required smarter prioritisation to decide ethically Equity risks persisted despite stakeholder input	Ethical allocation tools can improve fairness under scarcity, but implementation challenges (e.g., publicity and equity risks) remain	Program‑focused tool may miss individual patient needs Access inequities may exclude patients; the tool is not widely publicised
[41]	Explore primary healthcare managers’ experiences of DM during COVID‑19	Managers felt lonely in DM and stretched to limits, yet proud/resilient Increased demands and reduced control worsened psychosocial conditions Effort–reward imbalance heightened stress risk	Leadership support systems are needed; humane and just leadership may strengthen future crisis response	Volunteer bias; interview guide not manager‐specific May under‐capture those under the greatest strain and contextual complexity
[42]	Describe challenges in the US burn care infrastructure and crisis management during COVID‑19	Command teams described episodes shaped by organisation, adaptation, shared picture, assumptions and analysis Burn care capacity is limited and vulnerable to surge/mass casualty events Observation wards improved throughput and reduced unnecessary admissions	Resilient, adaptive systems and clearer integration of burn care into emergency plans can improve crisis care	Resource constraints and coordination gaps across partners Local plans and literature scope may omit aspects of burn response
[43]	Reflect on public leadership during crises and lessons from COVID‑19	Crisis required rapid decisions, clear communication and innovation Public health leaders faced political roles and complex social/economic pressures, and only reliable data can improve their decisions Leadership responses influenced organisational survival	Future readiness requires adaptable governance, technical competence, and empathetic communication	Public health professionals often lacked political experience/training Limited participation/visibility; lessons still evolving with changing crisis conditions
[44]	Examine knowledge translation exchange (KTE) and evidence‑informed policymaking among Iranian decision‑makers during COVID‑19	Ten themes on evidence use; knowledge brokers and KTE tools were important Barriers included negative beliefs, cultural obstacles, and limited time Research outputs were often underused in government decisions	Purposive KTE strategies and activated knowledge brokers can strengthen evidence use during urgent crises	Some interviews by phone; limited participant time reduced depth Context‑specific qualitative findings require cautious transfer
[45]	Explore experiences of producing/using rapid evidence products for COVID‑19 DM	Rapid evidence products were highly valued for national DM Quick decisions are needed to address the crisis Trust and credibility were crucial; communication/website issues hindered use High workload requires skilled, adaptable teams	Rapid evidence synthesis is vital for preparedness; investment in capacity, planning and relationships is needed	Limited provider/user pool; some authors were participants (potential bias) No patient representatives; limited cross‑sector perspectives; workload unsustainable
[13]	Examine how crises shape strategic‑level DM processes in healthcare organisations	Crisis can centralise decision processes and broaden actor involvement Politicisation varied with CEO stakeholder collaborations Reliance on CEO perceptions and decisions suggests adapting to wider stakeholder views in crisis	Reflecting on crisis decision processes can support transition to a post‑crisis ‘new normal’ and improve implementation governance	Limitations not explicitly stated; implied single‑context focus and CEO‑centric perspective
[46]	Identify decision bottlenecks in Norway's COVID‑19 vaccine rollout and how central authorities can support local DM	Multiple bottlenecks emerged; some could be reduced by adjusting external constraints Coordination and shared situational awareness could support distributed improvisation Important roles existed beyond initial prioritisation criteria	Central support can ease local bottlenecks through coordination and information sharing while maintaining trust and autonomy	Restricted participant access, limited system view; few authority perspectives Bottleneck identification and deeper dynamics hard to capture; single‑coder approach
[47]	Assess how the UK National Health Service (NHS) decision protocols adapted from traditional to emergency processes during COVID‑19	Protocols shifted for speed while retaining core values (welfare, equity) Urgency modified principles; need better quality/usability of effectiveness data Organisational values guided decisions but required realignment in crisis	Crisis DM needs timely, clear processes and transparent communication about temporary procedural changes	Senior‑only focus; online survey snapshot limits representativeness Limited frontline/mid‑level views; scalability beyond crisis unclear
[48]	Analyse how politicisation, urgency, uncertainty and fear shape public health emergency DM	Four conditions strongly influenced crisis decisions and research conduct Biomedical focus can crowd out infrastructure/system solutions; equity concerns Urgency and fear complicate consent and ethical reasoning	Balance rapid biomedical action with long‑term equity and system strengthening; reassess research priorities under crisis pressures	Blueprints may overlook structural causes (e.g., poverty and instability) Crisis visibility can overshadow underlying drivers; moral uncertainties in trial ethics
[49]	Identify factors enabling a Taipei hospital to manage SARS‐CoV2 effectively	Five survival factors: timing, DM, implementation, communication and trust Successful management despite a high‑risk setting; training and supplies supported unity Administrative focus due to limited medical countermeasures at the time	Early action, trust, communication and thorough implementation underpin effective crisis management	No direct observation; nurse‑only interviews limited perspectives Staff fear and broader views were not deeply explored
[50]	Identify ethical issues faced by public health responders and recommend training/process improvements	Twenty‑four ethical issues across emergencies; communication with vulnerable groups most common Responders sensed dilemmas but lacked formal ethical frameworks Community input important; practical steps proposed	Integrating public health ethics training and structured processes can strengthen ethical DM in emergencies	Limited ethics training constrained issue identification Some barriers and cultural considerations were underexplored
[51]	Explore critical decisions during a terrorist mass‑casualty incident and how uncertainty/time pressure shape command decisions	Most critical decisions occurred in the first 30 min under high uncertainty Situation assessment was central and shaped by experience/knowledge Uncertainty peaked after early decisions, requiring later adjustment	Commanders need strong early situation assessment and rapid decision capacity, with flexibility as uncertainty changes	Recall bias; radio logs miss informal communication Descriptive focus on what decisions were made (less on how/quality); staging framework not standardised

Abbreviation: DM, decision‐making.

#### 2.5.1 Meta‐Ethnographic Synthesis

**FIGURE 1:**
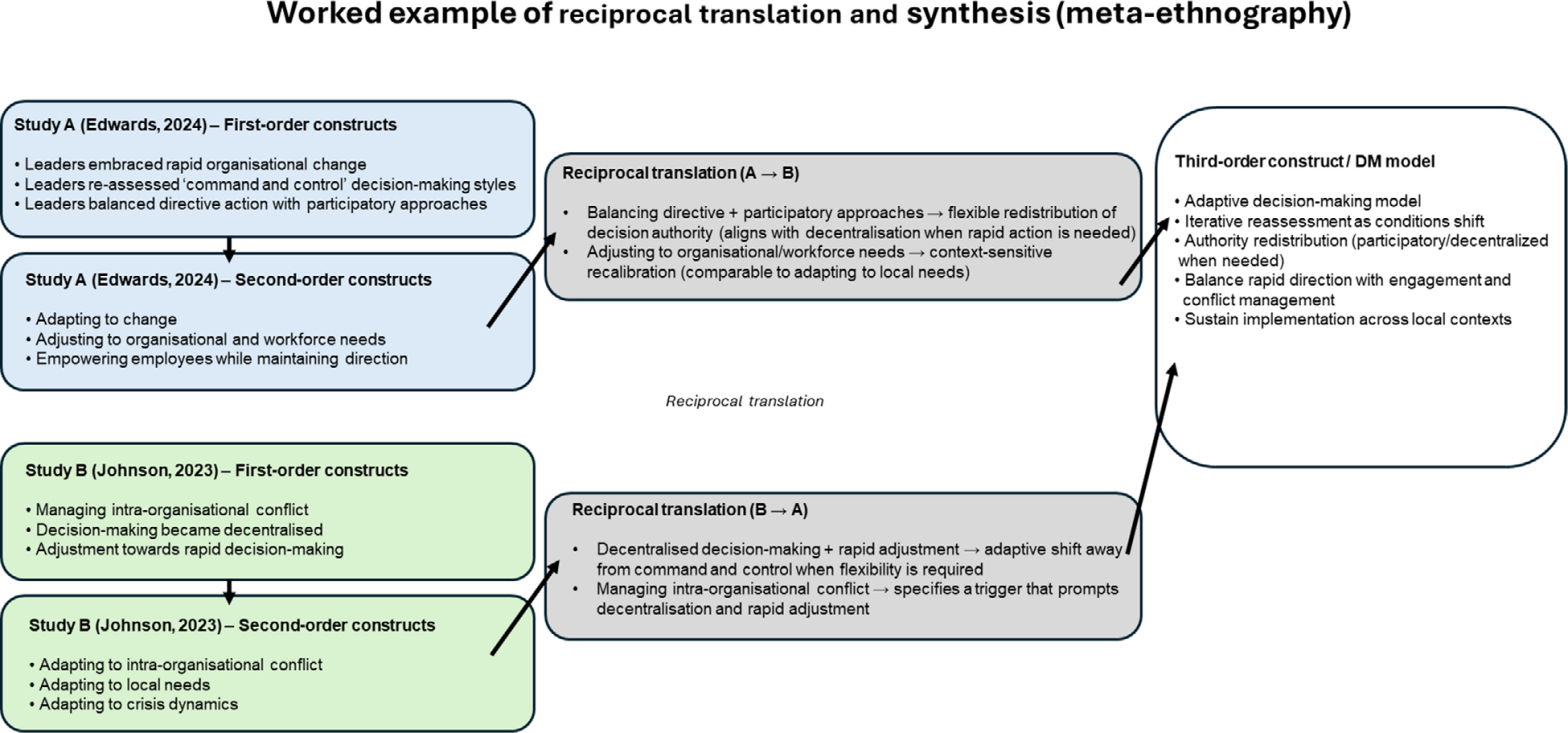
Illustrative worked example demonstrating the process of translation and synthesis across studies. DM, decision‐making.

The review adopted an interpretivist stance informed by critical realism, allowing for the exploration of both the meanings attributed to DM and the structural mechanisms that shape these processes under crisis conditions [52, 53, 54]. Data synthesis followed Noblit and Hare's seven‐step meta‐ethnography [26]. First‐order constructs (participants’ accounts) and second‐order constructs (primary authors’ interpretations) were extracted and compared across studies. Through reciprocal translation, conceptually similar constructs were translated into one another to develop third‐order constructs (review authors’ interpretations), which were then organised into DM models and prioritised decision factors. Figure 1 presents an illustrative worked example of the meta‐ethnographic synthesis, showing how first‐order constructs, second‐order interpretations, and third‐order interpretations were progressively translated across studies. By visually linking the analytic stages to the final synthesis, the figure enhances transparency and demonstrates how extracted qualitative evidence informed the identification of DM models and prioritised factors, whereas the broader translation matrix of DM and the full matrix of prioritised factors influencing DM are presented in Supporting Information Files 5 and 6, respectively.

## 3 Results

### 3.1 Overview

**FIGURE 2:**
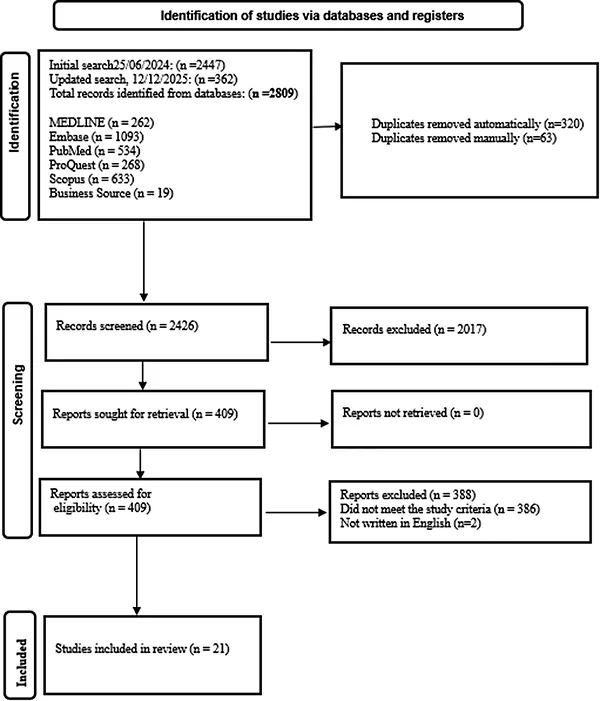
PRISMA 2020 flow diagram [24].

As shown in the PRISMA 2020 flow diagram (Figure 2), the searches retrieved 2809 records. After removing duplicates (*n* = 383), a total of 2426 records were screened by titles and abstracts, followed by a full‐text review of 409 papers. Twenty‐one studies met the review's inclusion criteria. Figure 2, therefore, provides a transparent account of the study selection process, showing how the initial evidence base was narrowed through duplicate removal, title and abstract screening and full‐text assessment. This supports the reproducibility of the review process and clarifies the basis on which the final studies were included. The completed PRISMA 2020 and eMERGe checklists are provided in Supporting Information Files 7–9. No studies were excluded on the basis of the quality appraisal (Supporting Information File 3).

### 3.2 Study Characteristics

**FIGURE 3:**
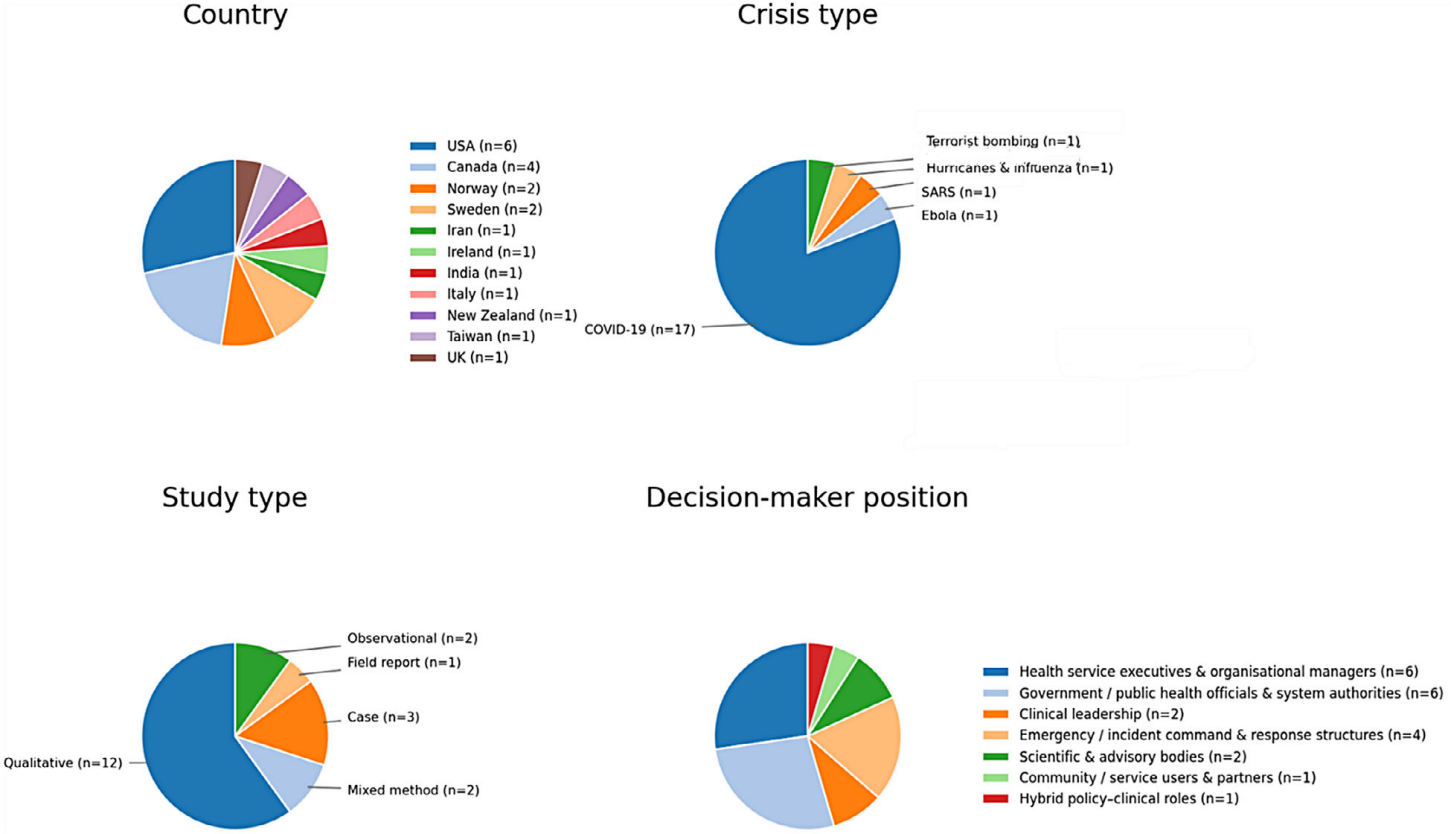
Included studies’ characteristics (country, type of public health crisis, study type and decision‐maker positions).

As shown in Figure 3, the included studies were concentrated in a small number of countries, predominantly the United States and Canada, with limited representation across other jurisdictions. Figure 3 contextualises the evidence base by displaying the distribution of included studies according to country, type of public health crisis, study type and decision‐maker position. This visual summary helps explain why the synthesis is strongly shaped by high‐income health system settings and by evidence generated during the COVID‐19 era. This concentration suggests that the available evidence is shaped largely by higher income health systems with relatively greater access to formal governance structures, surveillance systems and decision‐support capacity. Publications spanned from 1962 to 2025, although the distribution was heavily weighted toward the COVID‐19 era. The dominance of COVID‐19 studies indicates that current knowledge about crisis DM is strongly influenced by prolonged pandemic conditions, whereas other crisis types, including man‐made and natural events, were rarely represented. Decision‐maker roles were primarily positioned at senior organisational and system levels, including executive, managerial, government and public health roles, whereas community‐embedded and hybrid policy‐clinical roles were least represented.

### 3.3 Models of DM

**FIGURE 4:**
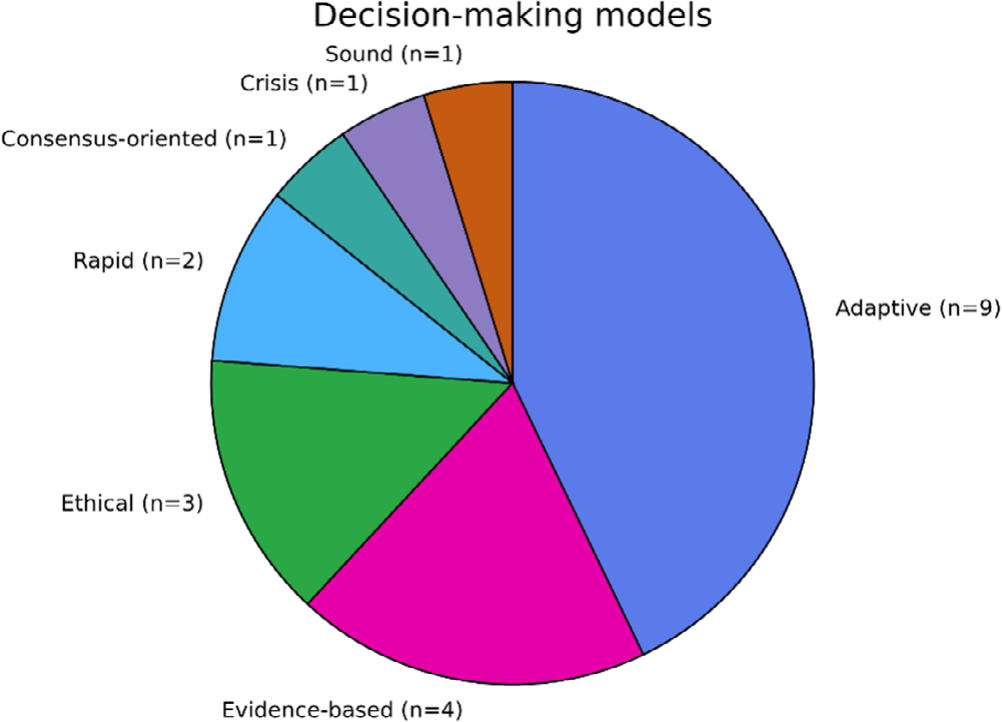
Distribution of decision‐making models across the included studies.

Across the studies, seven distinct DM models were identified (Table 2 and Figure 4). Table 2 shows how first‐order constructs from the included studies were translated into second‐order interpretations and then synthesised into third‐order DM model categories. Figure 4 complements this table by showing the distribution of these models across the included studies, making visible the predominance of adaptive and evidence‐based DM models. Adaptive (*n* = 9) and evidence‐based (*n* = 4) models were most frequently represented, whereas ethical (*n* = 3), rapid (*n* = 2), consensus‐oriented (*n* = 1), crisis (*n* = 1) and sound (*n* = 1) models were less common. Figure 4 visually demonstrates the predominance of adaptive and evidence‐based DM models across the included studies. This distribution supports the interpretation that public health crisis DM was often characterised by the need to respond flexibly to changing conditions while incorporating emerging evidence. The lower frequency of ethical, consensus‐oriented, crisis, rapid and sound DM models suggests that these approaches were more context‐specific or more visible during particular phases of crisis response. The identified DM models are not mutually exclusive; rather, they cluster into three broader functional orientations: adaptive–analytic (adaptive‐ and evidence‐based DM models), normative–value‐driven (ethical and sound DM models) and action‐oriented (rapid and crisis DM models). For the included studies, decision‐makers frequently combined or shifted between these orientations as the public health crisis conditions evolved. Adaptive and evidence‐based DM models were often used in tandem, reflecting the need to act under uncertainty while continually incorporating emerging information. Ethical and sound DM models were typically invoked to guide deliberation when value conflicts or accountability concerns arose but were constrained by time pressure during acute crisis phases. Rapid and crisis DM models were most evident in early or high‐severity phases of a public health crisis, where immediate action outweighed extended deliberation (see Tables 2 and 3 for the synthesis of themes). The broader translation matrix is provided in Supporting Information File 5.

**TABLE 2:** Mapping of themes to decision‐making (DM) models.

References	First‐order constructs (as stated in the study)	Second‐order constructs (authors’ interpretation)	Third‐order construct
[32]	Leaders realised the importance of embracing and capitalising on their organisations’ rapid change Leaders perceived an increased need to adapt to the requirements of their organisations and people and frequently reassessed traditional ‘command‐and‐control’ DM styles Leaders balanced being directive to move rapidly with participatory approaches to maintain staff engagement	Adapting to change Adjusting to organisational and workforce needs Empowering employees while maintaining direction	Adaptive model; iterative adjustment to shifting conditions, balancing control with participation
[33]	Managing intra‐organisational conflict was essential for decision‐makers DM became decentralised, with an adjustment toward rapid DM	Adapting to intra‐organisational conflict Adapting to local needs Adapting to crisis dynamics	Adaptive model; flexible, decentralised DM responsive to conflict, local variation and evolving crisis conditions
[34]	Supporting the values that guide ethical behaviours within the organisation Promoting an ethical mindset and supporting ethical DM at all organisational levels	Foregrounding an ethical approach during a crisis	Ethical model; value‐driven DM supported through organisation‐wide ethical culture and practice
[35]	Participants reported no specific DM tool during the emergency and instead relied on a consensus‐based DM process	Using consensus to avoid the risks of unilateral DM during a crisis	Consensus‐oriented model; collective agreement used to build legitimacy and reduce unilateral risk under emergency conditions
[36]	Analysing the crisis DM process using legal authority, meta‐leadership and decision intelligence Highlighting the value of decision intelligence in public health crisis management	Evaluating risks and potential outcomes under formal authority	Crisis model; DM; urgent action, high uncertainty, high stakes and imperfect preparedness, with a need to choose quickly while managing trade‑offs
[37]	Seven factors influenced DM Provided a qualitative description of transition DM in care	Adapting to guidance Adapting to change	Adaptive model; adjustment to evolving guidance and changing system conditions during transition phases
[38]	Created reproducible and scalable digital health dashboards for DM Delivered real‐time big data to control COVID‐19 risks and maintain public services Developed scalable dashboard systems for tracking epidemics and pandemics Democratised data ownership at individual and jurisdictional levels	Use of assistive technology Emphasis on information availability	Evidence‐based model; DM enabled by real‐time analytics and decision‐support tools
[39]	Decision‐makers faced a high volume of data not translated into user‐friendly reports and limited data on readiness and response actions Decision‐makers preferred evidence briefs with clear titles, target audiences, actionable checklists/graphics and implementation recommendations Respondents preferred crisis‐related sources of data	Evidence must be clear and usable Decision quality depends on evidence presentation Preference for reliable information sources	Evidence‐based model; emphasis on evidence usability, clarity and credibility to support actionable decisions under pressure
[40]	Accountability for reasonableness (A4R) was presented as an ethical DM method with broad application potential Routine re‐evaluation of plans was used to align with ethical principles and balance competing tasks	Application of an ethical framework Maintaining an ethical approach during crisis	Ethical model; explicit ethical framework and iterative ethical review guide decisions across crisis phases
[41]	Leaders described feeling alone in DM and pushed to the limit High workload due to shifting routines, new tasks and high sick leave rates increased pressure	Adapting under isolation Adapting to workload and routine change	Adaptive model; coping and adjustment under workforce strain, disrupted routines and role expansion
[42]	Organisational issues influenced the DM process Adaptation strategies were used to overcome restrictive organisational factors Limited knowledge affected DM and tactics to improve situational awareness	Adapting to obstructive organisational constraints Adapting under limited knowledge	Adaptive model; flexible workarounds to organisational barriers and information gaps to maintain situational awareness
[43]	Fast evidence products were essential for DM during the epidemic Highly competent personnel were required to produce rapid evidence options	Reasoned DM supported by knowledge sharing	Evidence‐based model; quick evidence synthesis and dissemination capacity support timely, reasoned crisis decisions
[44]	Evidence was used in a health crisis to inform DM	Evidence is used under crisis conditions	Evidence‐based model; explicit reliance on evidence to guide decisions under crisis constraints
[45]	Supported public health specialists in handling crises Highlighted rapid DM	Rapid decisions are positioned as necessary during a crisis	Rapid model; time‐critical DM prioritising speed and responsiveness in acute phases
[13]	CEOs changed how decisions were made as the crisis evolved	Decision processes adapted as the crisis evolved	Adaptive model; decision approach shifts across phases as conditions and demands change, and adapts to wider stakeholder views in crisis
[46]	Five decision bottlenecks emerged as the crisis developed	Adaptation was required to move through decision bottlenecks	Adaptive model; iterative problem‐solving is used to overcome bottlenecks as the crisis trajectory unfolds
[47]	‘Protocols were modified during the pandemic’	Flexibility replaced rigid processes and protocols	Adaptive model; protocol modifications reflect flexibility and learning, replacing rigid adherence to process
[48]	Politicisation, ambiguity, urgency and fear shaped decisions about the global health issue	Adapting to surrounding conditions	Adaptive model; decisions shaped by volatile context
[49]	Identified critical survival factors during the SARS crisis: effective timing, execution, interaction and confidence	Careful, structured DM	Sound model; deliberate timing and structured execution support confidence and coordination in‐crisis response
[50]	Recognised ethical concerns in public health emergencies and made feasible suggestions Respondents reported no formal training in public health ethics Recommendations included training in public health ethics to improve ethical capability	Addressing ethical issues in decisions Need for greater ethical capability in crisis management	Ethical model; ethical constraints and capability gaps highlight the need for structured ethics support and training
[51]	The ambulance commander made the most essential judgements within the first 30 min Personal experiences with large‐scale incidents influenced scenario evaluation	Quick and critical decisions Personal preferences and experience effects (experience bias)	Rapid model; time‐critical action (rapid) combined with situational adaptation shaped by experiential judgement (adaptive)

**TABLE 3:** Synthesis of factors prioritised in decision‐making (DM) during a public health crisis.

References	First‐order	Second‐order	Third‐order (prioritised factor)
[32]	Directors reported a greater desire to detect and adjust to their organisations and staff's requirements	Considering organisational needs and employees’ needs	Institutional requirements + urgency Prioritising organisational and workforce needs under time pressure to sustain operations
[33]	Handling organisational disputes is an essential component of DM	Prioritising resolution of internal conflict to facilitate the DM process	Intra‐organisational conflict Prioritising conflict resolution to enable coordinated action and clear decision pathways
[34]	Support ethical DM across all levels of the team	Prioritising ethics when making decisions	Ethical issues Foregrounding ethical considerations as a core criterion guiding crisis decisions across team levels
[35]	Necessity to have a step‐by‐step DM approach	Prioritising policies, procedures and frameworks	Knowledge Prioritising structured guidance (policies/procedures) as actionable knowledge to standardise decisions during a crisis
[36]	Relied on collective knowledge and intuition to issue shelter‐in‐place orders	Prioritising knowledge to make a decision	Knowledge Drawing on collective expertise and situational judgement as the primary basis for high‐stakes crisis directives
[37]	Seven factors appeared to guide DM	Guiding factors were prioritised and acted upon accordingly	Institutional requirements + risk + personal preferences and beliefs Balancing organisational constraints and risk appraisal with individual judgement in transition decisions
[38]	Developed replicable and scalable digital health dashboards for DM Big data were relayed in real time to manage COVID‐19 risks and public services	Prioritising available and reliable data to support decision‐makers	Knowledge Prioritising real‐time, reliable data infrastructure to strengthen situational awareness and support timely decisions
[39]	Decision‐makers preferred evidence summaries and sources of information in crisis decisions	Prioritising evidence and information to gain knowledge before deciding	Knowledge Prioritising synthesised evidence and credible information sources to inform choices under crisis uncertainty
[40]	Regular re‐evaluation of blueprints to address ethical principles and balance activities	Prioritising ethical principles while deciding	Ethical issues Maintaining ethical alignment through ongoing review and balancing of competing crisis activities
[41]	Primary leaders in healthcare must support staff capacity to develop with the role, adjust to shared objectives in a crisis and make sound judgements under stress	Sharing decisions	Teamwork and group structure Prioritising shared DM and alignment around common goals to maintain performance under stress
[42]	Organisational challenges affected the DM process	Considering organisational needs	Institutional requirements Prioritising organisational constraints and operational demands as key determinants of crisis decisions
[43]	Trust in evidence providers and skilled teams was crucial in DM	Skilled teams and trusted evidence enhance decision‐makers’ knowledge	Knowledge Prioritising trustworthy evidence and skilled analytic support to improve decision confidence and quality
[44]	Evidence is necessary, not just recommended	Evidence facilitates DM under crisis conditions	Knowledge Prioritising evidence as an essential input enabling defensible decisions during crisis conditions
[45]	Emphasises rapid DM and innovative thinking	Moving quickly in a crisis	Urgency Prioritising speed and responsiveness, with innovation used to sustain timely crisis action
[13]	Adjusted decision structures as information evolved	DM adapted to emerging information	Knowledge Prioritising evolving information as a driver for updating structures and choices across crisis phases
[46]	Centralised bodies have an opportunity to address external obstacles to alleviate frequent bottlenecks when they arise	Using prior knowledge to overcome external constraints	Knowledge Prioritising anticipatory understanding of external barriers to reduce bottlenecks and maintain decision flow
[47]	Balancing immediate response with long‐term commitments	Immediate response needs	Urgency Prioritising immediate response demands while negotiating longer term obligations during a crisis
[48]	Emphasis on interventions that raise ethical concerns	Avoiding the risk of clashing with ethics	Risk + ethical issues Prioritising mitigation of harm and ethical conflict when evaluating contentious interventions
[49]	Good control of timing for crisis management	Moving quickly in a crisis	Urgency Prioritising time control and rapid action as core requirements of effective crisis management
[50]	Identified moral challenges in health‐related situations and provided practical advice Respondents lacked official education in health‐related principles	Avoiding the risk of clashing with ethics	Risk + ethical issues Prioritising the reduction of ethical risk and moral harm, highlighting capability gaps requiring practical guidance
[51]	The ambulance commander made the most essential judgements within the first 30 min period	Moving quickly in a crisis	Urgency + risk Prioritising rapid action under severe time constraints while managing high‐stakes operational risk

#### 3.3.1 Adaptive DM Model

The adaptive DM model is the ability to adjust to a situational change thoughtfully and efficiently [55]. Nine studies indicated that the adaptive DM model was utilised during a crisis [13, 32, 33, 37, 41, 42, 46, 47, 48]. The studies employed adaptive DM models to respond effectively to evolving circumstances, manage intra‐organisational conflicts, address workload pressures, overcome obstructive organisational constraints, navigate contextual challenges and accommodate individual preferences. An adaptive DM model is useful in such contexts, as it enables decision‐makers to continuously receive updated data, assess conditions and adjust strategies as needed [56]. Pettersson illustrates adaptive DM in practice, where decision‐makers employed adaptation strategies to navigate restrictive organisational factors and compensate for limited knowledge by implementing tactics that enhanced situational awareness [42]. This demonstrates flexible, iterative adjustment through modification to decision processes and operational tactics in response to organisational barriers and information gaps during crisis conditions.

#### 3.3.2 Evidence‐Based DM Model

The evidence‐based DM model refers to the process of making better decisions by combining critical thinking with evidence‐based reasoning [57]. Four studies [38, 39, 43, 44] utilised the evidence‐based DM model, emphasising that decision quality depends on the availability and clarity of information. Evidence must be explicit and accessible, even under crisis conditions, as reasoning grounded in reliable data enhances decision accuracy. Furthermore, decision‐makers are encouraged to leverage advanced technologies to support informed, timely responses during a crisis. For instance, Bhat reports that rapid evidence products enabled by skilled personnel and knowledge sharing supported timely, reasoned decisions during a pandemic [43].

#### 3.3.3 Ethical DM Model

The ethical DM model is the process of making a decision that adheres to ethical principles [58]. Three studies [34, 40, 50] employed ethical DM models that emphasised the importance of upholding organisational values that guide ethical behaviour, integrating ethical principles into decision processes and balancing competing tasks. These models place greater emphasis on the ethical dimensions of crisis management. Johnson‐Cornett reported that values‐driven DM was strengthened through an organisation‐wide ethical mindset and support for ethical practice at all levels during crisis conditions [34]. This demonstrates how ethical DM is operationalised through organisation‐wide ethical culture and everyday practice, rather than relying solely on individual judgement.

#### 3.3.4 Rapid DM Model

The rapid DM model is the process of making well‐informed decisions quickly and effectively [59]. Two studies highlighted the usefulness of rapid DM model during a crisis [45, 51] when there was a need for rapid and critical actions to address urgent challenges during crises. Notably, Rimstad reports that an ambulance commander made key judgements within the first 30 min, with scenario evaluation shaped by prior large‐scale incident experience [51].

#### 3.3.5 Consensus‐Oriented DM Model

A consensus‐oriented DM model is a collaborative approach to problem resolution that helps teams reach conclusions that satisfy all stakeholders [60]. One study [35] identified the use of the consensus‐oriented DM model. Barishansky demonstrated that, in the absence of a formal DM tool, leaders relied on consensus to reduce unilateral risk and strengthen legitimacy during the emergency [35].

#### 3.3.6 Crisis DM Model

One study indicated that the crisis DM model is ideal to employ during a crisis as it provides a collaborative approach to risk assessment and potential outcomes [36]. The study examined the crisis DM process through the application of legal authority, meta‐leadership principles and decision intelligence, highlighting the critical role of informed and strategic reasoning in managing public health crises. Aragón examined crisis DM under formal legal authority, demonstrating the use of decision intelligence to evaluate risks, anticipate potential outcomes and make urgent decisions under conditions of high uncertainty and significant stakes [36].

#### 3.3.7 Sound DM Model

The sound DM model involves carefully weighing all options, possible consequences and the viewpoints of others [61]. One study [49] demonstrated the utility of sound DM model during a public health crisis through careful deliberation, effective timing, thorough execution, structured interaction and confidence in decision processes. Tseng found that during SARS, effective timing, execution, structured interaction and confidence were critical, illustrating sound DM as deliberate, structured DM that strengthens coordination in crisis response [49].

#### 3.3.8 Contextual Influence on DM

Model selection varied across crisis contexts. In prolonged crises such as COVID‐19, adaptive and evidence‐based models predominated, reflecting repeated cycles of DM and incremental learning as evidence evolved [33, 42, 44]. By contrast, in short, high‐impact events such as terrorist incidents and natural disasters, rapid and crisis‐oriented models were more prominent, prioritising immediacy over iterative analysis [51].

Contextual differences were also evident across health system settings. A study from an LMIC setting emphasised adaptive DM under constraints of limited data, workforce shortages and regulatory ambiguity [13], whereas HIC settings more frequently reported evidence‐based approaches supported by formal surveillance systems and digital decision‐support tools [12, 38]. These findings indicate that model selection is shaped not only by the duration and type of the crisis, but also by systemic capacity and governance structures for responding to it.

The decision‐makers’ selection and application of the DM model reflected a continuous negotiation among information availability, urgency and structural constraints (Table 2). From a systems thinking perspective, adaptive and evidence‐based models act as stabilising mechanisms, enabling decision‐makers to respond to feedback while maintaining analytical coherence. In crisis governance terms, these models support sensemaking and coordinated action under conditions of uncertainty.

More deliberative approaches, such as sound or consensus‐oriented models, were not abandoned but were conditionally sidelined during acute phases when speed was essential. Their use became more visible during stabilisation or recovery phases, underscoring that model selection is dynamic rather than fixed. These findings suggest that effective crisis DM depends less on adherence to a single model and more on leaders’ capacity to switch between models as system conditions change.

### 3.4 Prioritised Factors Influencing DM

#### 3.4.1 Overview

**FIGURE 5:**
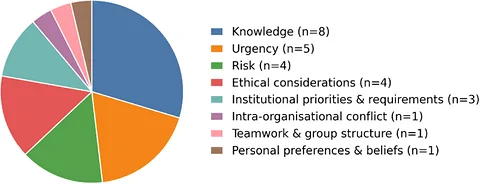
Prioritised factors influencing decision‐making.

Eight prioritised factors were identified, with frequencies reflecting their occurrence across the included studies. Knowledge (*n* = 8) and urgency (*n* = 5) were most frequently prioritised, followed by risk (*n* = 4); ethical issues (*n* = 4) and institutional priorities; and requirements were also reported (*n* = 3). Intra‐organisational conflict (*n* = 1), teamwork and group structure (*n* = 1) and personal preferences and beliefs were reported infrequently (*n* = 1). Figure 5 visually summarises the relative prominence of the prioritised factors, showing that knowledge and urgency were the most frequently identified influences on DM. This pattern suggests that crisis decisions were commonly driven by the need to act quickly while relying on the best available information. These frequencies should be interpreted as indicators of salience under crisis conditions rather than as a fixed hierarchy of importance. Knowledge and urgency acted as foundational conditions enabling action and shaping how risk, ethics and institutional constraints were interpreted. Together, they formed a dual entry point into decision processes, with other factors becoming more visible after initial action pathways were established. The lower frequency of ethical issues, institutional requirements, teamwork, intra‐organisational conflict and personal preferences does not necessarily indicate that these factors were unimportant, but rather that relational, organisational and value‐based dimensions remain less explicitly examined in the included literature.

Table 3 presents the meta‐ethnographic synthesis of prioritised factors influencing DM across the included studies, showing how first‐order constructs were translated into second‐order interpretations and then into third‐order prioritised factors. The broader translation matrix is provided in Supporting Information File 6.

#### 3.4.2 Knowledge

Eight studies indicated that decision‐makers prioritise knowledge when making decisions [13, 35, 36, 38, 39, 43, 44, 46]. Knowledge is crucial to understanding the crisis's nature, origin and possible effects [62]. In these studies, knowledge included information, skills, expert advice and assistive technology. Obtaining precise, up‐to‐date information is a top priority for decision‐makers to assess situations thoroughly and prefer assistive technology to support informed decisions [38]. Knowledge of the legal frameworks, regulations and ethical standards also assisted in guiding DM during crises [38].

#### 3.4.3 Urgency

Crises require prompt action to limit damage and avoid further escalation [32, 51]. Five studies [32, 45, 47, 49, 51] reported that decision‐makers prioritised urgent factors, such as the need to move quickly through the crisis, respond immediately and reach decisions even when data were incomplete when making time‐critical choices.

#### 3.4.4 Risk

Exploring risks is one of the first priorities for decision‐makers [37], and four studies found that decision‐makers prioritise the likelihood and magnitude of risks over other factors [37, 48, 50, 51]. Risk‐taking in the face of significant ambiguity played an important role in the DM process [51].

#### 3.4.5 Ethical Considerations

Four studies prioritised ethical considerations by re‐evaluating decisions during the DM process to reflect ethical standards and balance competing priorities and activities when making decisions [34, 40, 48, 50]. Prioritising actions that protect people's rights, privacy, safety and equitable treatment was seen as paramount to decision‐makers [50].

#### 3.4.6 Institutional Priorities and Requirements

According to three of the studies, it is more important to recognise and accommodate the demands of both the organisation and its people when making decisions during a crisis [32, 37, 42]. The studies indicated that organisational challenges and unmet employee needs serve as obstacles that should be considered first [37].

#### 3.4.7 Intra‐Organisational Conflict

One study indicated that decisions should be made during times of crisis after internal conflicts within the organisation have been resolved [33]. Group coherence and decisiveness without conflict are required to deliver instructions, updates and key information to decision‐makers [33].

#### 3.4.8 Teamwork and Group Structure

Another study highlighted the importance of prioritising teamwork and individuals’ capacity to evolve to the task at hand and align with shared aims during a crisis [41]. Leaders need to balance group structure and dynamics to enhance the employees’ participation in the DM process [41].

#### 3.4.9 Personal Preferences and Beliefs

One study [37] indicated that decision‐makers’ personal preferences and beliefs can consistently frame DM. Making decisions with integrity and coherence is encouraged when decision‐makers prioritise options that are consistent with their deeply held values. For instance, Carbone demonstrated this by highlighting the need to recognise older adults’ personalities and include their preferences in transition decisions during a crisis [37].

#### 3.4.10 Contextual Variation in Prioritised Factors

Contextual variation in prioritised factors was evident across settings. In HIC contexts, knowledge was often operationalised through formal data systems, modelling tools and advisory structures, enabling evidence‐based DM [12, 32, 33, 47]. In contrast, studies from LMIC settings described knowledge as situational awareness derived from experiential judgement, informal networks and rapidly evolving local information [13, 43, 44]. Similarly, urgency in resource‐constrained contexts was often compounded by workforce shortages and supply limitations, amplifying risk tolerance and constraining ethical deliberation [41, 48, 51].

#### 3.4.11 Interpretation: Urgency and Knowledge as System Regulators

Across crisis contexts, urgency and knowledge functioned as interdependent regulators of DM rather than competing priorities. From a systems perspective, they operate through a feedback loop: Urgency accelerates action, whereas knowledge moderates and recalibrates decisions by reducing uncertainty. Importantly, urgency can constrain the use of knowledge, whereas available knowledge can reshape perceived urgency, influencing how decisions are made. Adaptive and evidence‐based models were commonly used to manage this interplay, enabling rapid action with iterative adjustment. This suggests that crisis DM is less about optimisation and more about maintaining system function under pressure. However, the predominance of studies from HIC (e.g., the United States and Canada) means this dynamic may differ in resource‑constrained contexts, where limitations in data, evidence and governance capacity may alter the balance between urgency and knowledge.

## 4 Discussion

Beyond describing the evidence base, the study characteristics indicate that DM models reported in the literature are shaped by both crisis type and institutional context. The predominance of COVID‐19 studies reflects prolonged, system‐wide crises characterised by sustained uncertainty, repeated decision cycles and evolving evidence, which necessitate adaptive and evidence‐based approaches. The concentration of studies in HIC suggests that most identified DM practices are embedded within comparatively well‐resourced health systems, where access to data, digital infrastructure and formal governance mechanisms is more likely. However, the inclusion of studies from LMIC introduces important variation, highlighting how constraints in information availability, regulatory capacity and organisational resources influence model selection and factor prioritisation. Together, these characteristics frame the synthesis as reflecting both context‐specific practices and cross‐cutting decision dynamics.

The figures further support this interpretation by linking the review process, evidence characteristics and synthesis findings. Figure 2 provides transparency on how the evidence base was narrowed through the PRISMA screening process, whereas Figure 3 contextualises the included studies by showing their concentration in particular countries, crisis types and decision‐maker roles. Figures 4 and 5 extend this interpretation by visually demonstrating the predominance of adaptive and evidence‐based DM models and the centrality of knowledge and urgency as prioritised factors. Together, these visual findings reinforce the argument that public health crisis DM is shaped by the interaction between rapidly changing conditions, available information and the need for timely action.

The findings depict DM in public health crises as dynamic and context‐responsive. Decision‐makers operate within complex adaptive systems, continually balancing urgency, available knowledge and institutional constraints rather than applying discrete, static models. From a systems thinking perspective, DM is best understood as a dynamic process shaped by feedback loops, resource limitations and evolving contextual pressures, rather than adherence to a single ‘best practice’ model [2, 3, 4].

These findings also have important implications for health systems’ resilience. Resilient health systems are not only those that absorb shocks but also those that can adapt, maintain essential functions and transform when exposed to crises [63, 64]. In this review, the predominance of adaptive and evidence‐based DM models suggests that resilience depends on leaders’ capacity to adjust decisions as conditions change while still maintaining analytic coherence and operational continuity. However, the findings also show that resilience is unevenly distributed across contexts. In well‐resourced systems, adaptive and evidence‐based decisions may be supported by formal data infrastructure, modelling capacity and established advisory mechanisms. In LMIC and other resource‐constrained settings, by contrast, resilience may depend more heavily on local knowledge, informal networks, experiential judgement and flexible use of limited resources. This indicates that crisis DM frameworks should avoid assuming that all health systems have equal capacity to generate, access or implement evidence during crises.

The predominance of the adaptive DM model reflects the dynamic, uncertain conditions of public health crises, which necessitate rapid responsiveness and explains why adaptive DM was the most common model reported in this review [65]. Adaptive DM enables learning and recalibration as evidence evolves, supporting resilience and more nuanced engagement with complex systems [65, 66]. Evidence‐based DM was the second most commonly used approach, consistent with leaders drawing on routine decision habits during crises, particularly given that many decision‐makers had clinical backgrounds and the clinical origins of evidence‐based DM [66, 67, 68, 69, 70]. However, the review highlights trade‐offs where adaptive DM may normalise constant recalibration without strong ethical or accountability safeguards, whereas evidence‐based DM may privilege formal evidence over contextual knowledge when high‐quality data are limited early in a crisis [65, 66, 67, 68, 69, 70].

Ethical DM models emerged as essential but structurally constrained [71]. Although ethical reasoning supports professional accountability, moral integrity and public trust, it was less prominent during acute crisis phases, where urgency and operational pressures dominated. This finding highlights a persistent tension: Although ethical deliberation is widely recognised as critical, existing crisis conditions and governance arrangements may limit its integration into real‐time decision processes. The findings, therefore, raise important questions about how ethical governance can be meaningfully embedded into crisis DM without impeding timely response.

The pattern of model selection observed in this review aligns closely with crisis governance theory [9, 72]. Decision‐makers consistently prioritised models that enabled rapid sensemaking, coordinated action and institutional control, often at the expense of deliberative or consensus‐oriented approaches [30, 73]. This reflects a governance reality in which legitimacy during crises is frequently maintained through decisiveness rather than inclusivity [74]. However, privileging speed and hierarchy may marginalise participatory DM and exacerbate power asymmetries, particularly in contexts where governance structures are weak or fragmented [75]. The findings thus expose a core governance dilemma: how to preserve legitimacy, coordination and accountability while responding under extreme time pressure and uncertainty.

This governance dilemma is particularly important for LMIC settings, where crisis decisions may be shaped by decentralisation, donor dependency, fragmented authority, limited regulatory capacity and constrained accountability mechanisms. The findings suggest that governance models based solely on centralised command may support rapid action, but they may also reduce local responsiveness and exclude community‐level knowledge. Conversely, participatory or consensus‐oriented governance may strengthen legitimacy and equity but can be difficult to operationalise when time pressure is extreme and institutional capacity is limited. A more context‐sensitive governance model is therefore needed, one that combines clear decision authority with mechanisms for local adaptation, community engagement and accountability. Such an approach would better reflect the realities of crisis management in LMIC health systems, where decisions are often filtered through relationships, organisational norms, resource constraints and power inequalities [76]. A critical contribution of this review is to show how DM models and prioritised factors are shaped by health system capacity and global inequality. Most included studies were conducted in HIC settings, where evidence‐based DM is supported by formal surveillance systems, modelling tools and established advisory structures. In contrast, studies from LMICs described DM grounded in situational awareness, experiential judgement and informal information networks.

Urgency was also experienced differently across contexts. In resource‐constrained settings, urgency was intensified by workforce shortages, supply limitations and fragile infrastructure, often increasing risk tolerance and constraining ethical deliberation. These dynamics raise important equity concerns, as leaders in LMICs and in rural and remote regions must make high‐stakes decisions under structural constraints that are largely absent in well‐resourced, urban, high‐income settings. This imbalance highlights the need for crisis DM frameworks that are context‐sensitive and equity‐oriented, rather than implicitly privileging the assumptions and capacities of high‐income or metropolitan systems. These findings also establish several priorities for future scholarly research. First, more empirical studies are needed to examine how DM models are selected, combined and adapted across different phases of public health crises. Second, future research should move beyond retrospective accounts and incorporate longitudinal, observational and real‐time approaches to capture how decisions are made as crises unfold. Third, scholars should give greater attention to under‐represented settings, particularly LMIC, rural and resource‐constrained health systems, where limited data infrastructure, workforce capacity and governance resources may shape DM differently. Finally, future studies should examine how equity, ethics, community participation and accountability can be embedded into crisis DM without delaying urgent action.

### 4.1 Policy and Practice Implications

The findings of this review have concrete implications for policymakers, public health practitioners and health system leaders responsible for crisis preparedness and response.

For policymakers, the results indicate that crisis DM is most effective when governance arrangements explicitly accommodate model switching across crisis phases. Policy frameworks should therefore clarify decision authority, escalation thresholds and accountability mechanisms in advance, enabling leaders to move between adaptive, evidence‑based and rapid DM approaches as conditions evolve. Embedding ethical oversight structures, such as rapid ethics advisory mechanisms, can help preserve legitimacy and public trust without delaying urgent action. In addition, policymakers should establish sustained partnerships with mathematical modellers, biostatisticians and other analytics experts to ensure that policy‐relevant questions can be addressed rapidly during crises and that the uncertainties underpinning analytic outputs are communicated clearly and transparently [77].

For public health practitioners and emergency response teams, recognising DM models as practical tools rather than abstract frameworks can improve operational effectiveness. Adaptive DM is particularly valuable during early and acute crisis phases, when evidence is incomplete and conditions change rapidly, whereas evidence‑based approaches become more influential as information stabilises. Training and simulation exercises should therefore focus not only on strengthening practitioners’ ability to assess contextual signals, such as urgency, uncertainty and resource constraints, but also on improving their capacity to work with modellers and analytics experts, interpret analytic outputs and understand their limitations in real time. Strengthening these translational capabilities may improve the use of policy‐relevant evidence during future public health emergencies [77].

For health systems and organisational leaders, the findings highlight the importance of strengthening system‑level decision infrastructure. Investments in interoperable data systems, clear communication pathways and workforce surge capacity can reduce the trade‑off between urgency and knowledge that constrains crisis decisions. In lower resource settings, prioritising flexible, low‑cost decision‑support mechanisms that integrate local and experiential knowledge may be more effective than replicating high‑income technological models. Across all contexts, leaders should prioritise institutionalising domestic advanced analytics capacity and collaborative data‐to‐decision pathways, rather than relying solely on ad hoc emergency arrangements. Building and sustaining these partnerships before crises occur may help DM bodies identify where analytic capacity needs to be strengthened and support analytics experts to produce more policy‐relevant evidence during emergencies. Aligning decision support with equity goals, including workforce protection, transparent communication and ethical review, may also help mitigate the disproportionate effects of crises on vulnerable populations [78]. For LMIC policymakers and health system leaders, the findings suggest that strengthening crisis DM should prioritise feasible, equity‐oriented and locally adaptable infrastructure rather than simply replicating HIC models. This includes investing in basic surveillance capacity, workforce protection, transparent communication channels, local evidence‐use mechanisms and community‐informed decision processes [22, 79]. Where advanced modelling or digital systems are not immediately feasible, decision support can still be strengthened through rapid evidence summaries, locally embedded knowledge brokers, scenario‐based planning and structured escalation pathways. These strategies may help reduce inequities by ensuring that crisis decisions account for the needs of underserved populations, rural communities and groups with limited access to services [22, 79].

## 5 Future Research Directions

Future research should generate stronger empirical evidence on real‐time DM during public health crises through interviews, observational approaches and longitudinal case studies with policymakers, health executives and emergency response teams. Particular priority should be given to empirical studies in LMIC and under‐represented settings to examine how governance capacity, resource constraints, equity priorities and local knowledge shape crisis DM in contexts where formal decision‐support infrastructure may be limited.

For scholars, priority areas include examining how DM models are selected and adapted across crisis phases and how contextual pressures shape those decisions, as well as considering the development of new DM models in line with the knowledge advancing from this review and the broader field. For stakeholders, these findings establish a clear research agenda focused on building, testing and implementing practical decision infrastructure, including real‐time decision support systems, phase‐specific protocols and scenario‐based training, to enable appropriate model use and improve coordination during crises.

## 6 Methodological Considerations and Limitations

This review adopted a qualitative meta‑ethnographic approach to synthesise decision‑making during public health crises. Although this enabled in‑depth interpretation of decision processes and contextual influences, several limitations should be noted. First, selection and publication bias may be present, as the review included only peer‑reviewed, English‑language studies. Relevant evidence from non‑English sources and grey literature (e.g., policy reports and governmental reviews) may, therefore, be under‑represented, particularly from LMIC. Second, the geographical distribution of included studies was skewed toward high‑income settings, which may limit the transferability of findings to resource‑constrained health systems with different governance structures, data infrastructure and workforce capacity. Third, limitations inherent to meta‑ethnography should be acknowledged. The synthesis process is interpretive, and the translation of concepts across studies relies on analytical judgement by the authors. Although systematic procedures and cross‑checking were used to enhance rigour, some degree of subjectivity in developing third‑order interpretations is unavoidable.

Finally, heterogeneity across study designs, crisis types and participant roles means that the frequency with which models or factors were identified should be interpreted as indicative of salience within the literature rather than hierarchical importance. Despite these limitations, the review provides a theoretically informed synthesis of crisis decision‑making, while highlighting the need for further empirical research in diverse and under‑represented contexts.

## 7 Conclusion

This review advances understanding of decision‑making during public health crises through an integrated meta‑ethnographic synthesis of decision‑making models and the factors shaping their real‑world use. The findings show that crisis decision‑making is a dynamic, system‑embedded process in which health leaders continually balance urgency, knowledge availability, risk and institutional constraints. The predominance of adaptive and evidence‑based models reflects the need for flexibility and analytical grounding in fast‑moving, high‑pressure conditions, whereas the more limited use of ethical and deliberative approaches signals enduring challenges in governance, transparency and accountability.

A key contribution is the identification of urgency and knowledge as interdependent regulators of decision‑making rather than isolated influences. This interaction helps explain why models are selected, combined or adapted across crisis phases and varying system capacities and why these dynamics differ according to governance arrangements, resource availability and global inequities.

From a policy and practice perspective, strengthening preparedness requires more than technical tools. It demands adaptive decision capacity, clearer decision rights and escalation pathways and equity‑oriented safeguards that enable timely yet accountable action. Looking forward, decision‑making models must evolve into a ‘new normal’ of institutionalised crisis‑ready approaches embedded in routine systems, training and performance expectations. Future scholarship should build on these findings by examining how crisis DM models operate across diverse health system contexts, particularly in LMIC and resource‐constrained settings, and by testing practical frameworks that integrate resilience, equity, ethics and accountability into real‐time decision processes. Building and sustaining this capability should be treated as a core workforce priority. Crisis decision competence should be deliberately transferred through scenario‑based training, mentorship and decision infrastructure to the next generation of health leaders to meet future public health emergencies with greater effectiveness and equity.

## Author Contributions

Ehmaidy Al Qaf'an conducted the database searches and screened titles and abstracts, together with Stewart Alford. Decisions on paper inclusion were made collaboratively by Ehmaidy Al Qaf'an, Stewart Alford and Holly A. Mack. Ehmaidy Al Qaf'an was responsible for writing the manuscript, whereas Stewart Alford, Holly A. Mack and David Lim contributed to the editing and review process, ensuring the manuscript's accuracy and clarity. Each author played a vital role in the development and finalisation of this systematic review.

## Funding

The authors have nothing to report.

## Ethics Statement

The Western Sydney University Human Research Ethics Committee (EX2023‐18) exempted this systematic review from human research ethics.

## Conflicts of Interest

The authors declare no conflicts of interest.

## Reflexivity

The review reflects the authors’ professional backgrounds in public health management and practice, as well as their direct involvement in COVID‐19 and other public health crisis responses. This insider perspective shaped the conceptualisation of DM as a context‐dependent and collaborative process rather than a purely technical one. The authors acknowledge that their experience influenced the focus of the research, which was guided by leading teams and organisational responses before, during and post‐public health crises and by the teaching of disaster management and disaster medicine. Reflexivity was applied throughout the synthesis process via independent screening, iterative interpretation, and critical self‐reflection to minimise bias and enhance trustworthiness. Descriptive summaries were used for transparency but did not determine the interpretive analysis, which focused on comparing and integrating concepts across studies.

## Supporting information




**Supplementary File 1**: Search concepts and search terms.
**Supplementary File 2**: Search history.
**Supplementary File 3**: Critical appraisal of included studies.
**Supplementary File 4**: Data extracted.
**Supplementary File 5**: Meta ethnographic analysis of decision‐making (DM) models, presented as a translation matrix with reciprocal and refutational notes.
**Supplementary File 6**: Meta‐ethnographic analysis of prioritised factors influencing decision‐making (DM): translation matrix with reciprocal/refutational notes.
**Supplementary File 7**: PRISMA 2020 main checklist.
**Supplementary File 8**: PRISMA 2020 abstract checklist.
**Supplementary File 9**: eMERGe checklist.

## Data Availability

The data that support the findings of this study are available from the corresponding author upon reasonable request.
