# Supplementary material for: Decision‐Making in Public Health Crises: A Qualitative Systematic Review and Meta‐Ethnography
Source: Public Health Chall. 2026 Sep 15;5(3):e70358. doi: 10.1002/puh2.70358 (PMC13577831; doi:10.1002/puh2.70358)
Supplement: Supplementary file 1 [file PUH2-5-e70358-s001.docx]

# Supplementary Files

- Supplementary File 1: Search concepts and search terms
- Supplementary File 2: Search history
- Supplementary File 3: Critical appraisal of included studies
- Supplementary File 4: Data extracted
- Supplementary File 5: Meta ethnographic analysis of decision making (DM) models, presented as a translation matrix with reciprocal and refutational notes
- Supplementary File 6: Meta-ethnographic analysis of prioritised factors influencing decision-making (DM): translation matrix with reciprocal/refutational notes
- Supplementary File 7: PRISMA 2020 main checklist
- Supplementary File 8: PRISMA 2020 abstract checklist
- Supplementary File 9: eMERGe checklist

# Supplementary File 1: Search concepts and search terms

| **Search concepts** | **Search terms** |
| --- | --- |
| Healthcare leaders/ managers | Healthcare leader *  Healthcare executive*  Healthcare administrator*  Medical leader*  Clinical leader*  Hospital administrator*  Health system leader*  Healthcare manager* |
| Experiences or reflection on healthcare leadership | Leadership strategy*  Strategies in leadership  Leadership approach*  Effective leadership  Successful leadership  Leadership style*  Strategic leadership  Leadership tactic*  Innovative leadership  Adaptive leadership  Transformational leadership  Situational leadership  Leadership [Mh]  Experience*  Reflect* |
| Decision-making | Emergency* decision-making  Disaster* decision-making  Healthcare crisis* decision*  Medical emergency* decision*  Hospital crisis* management  Decision strategy* in crises*  Decision process* during emergency*  Decision-making in critical situations*  Decision-making process*  Decision process*  Decision-making mechanism*  Decision modelling  Decision-making pathway*  Medical decision-making  Healthcare decision-making  Clinical decision process*  Decision making [Mh]  Mental process* [Mh] |
| Healthcare, public health, or population health. | Hospital*  Community*  Medical care  Medical industry*  Public health  Population health  Health [Mh]  Organi?ations [Mh]  Delivery of health care [Mh]  Tertiary healthcare [Mh]  Primary health care [Mh]  Patient* care management [Mh] |
| Crisis type | Global health crisis*  Public health crisis*  Public Health emergency*  Public health disaster*  Crisis in public health  Disease outbreak*  Pandemic*  Epidemic*  Infectious disease*  Bioterrorism and public health  Environmental health crises* |
| In-crisis or during a crisis | Emergency response*  Emergency* preparedness  Emergency management  Disaster response*  Disaster preparedness  Disaster management  Healthcare emergency response*  Medical emergency preparedness  Hospital emergency management  Crisis response*  Crisis preparedness  Crisis management  Crisis management  Emergency management  Disaster management  Crisis  Critical care [Mh] |

# Supplementary File 2: Search history

**Ovid MEDLINE** **search 24^th^ January 2024**

|  | **Searches** | **Results** |
| --- | --- | --- |
| 1 | Health?care.mp. or exp Healthcare Disparities/ or exp "Healthcare Failure Mode and Effect Analysis"/ | 386564 |
| 2 | Health?care.mp. | 386564 |
| 3 | Clinical.mp. | 5184900 |
| 4 | Medical.mp. | 1980090 |
| 5 | Hospital*.mp. | 1990536 |
| 6 | Health system*.mp. | 82099 |
| 7 | primary health*.mp. | 120434 |
| 8 | public health*.mp. | 444144 |
| 9 | population health*.mp. | 18364 |
| 10 | community health*.mp. | 93425 |
| 11 | (health* adj2 organi?ation*).mp. [mp=title, book title, abstract, original title, name of substance word, subject heading word, floating sub-heading word, keyword heading word, organism supplementary concept word, protocol supplementary concept word, rare disease supplementary concept word, unique identifier, synonyms, population supplementary concept word, anatomy supplementary concept word] | 168492 |
| 12 | patient care management.mp. | 5304 |
| 13 | Emergency department*.mp. | 129341 |
| 14 | 1 or 2 or 3 or 4 or 5 or 6 or 7 or 8 or 9 or 10 or 11 or 12 or 13 | 8382798 |
| 15 | Leader*.mp. | 124879 |
| 16 | Leadership strateg*.mp. | 271 |
| 17 | (Strateg* adj1 leadership).mp. [mp=title, book title, abstract, original title, name of substance word, subject heading word, floating sub-heading word, keyword heading word, organism supplementary concept word, protocol supplementary concept word, rare disease supplementary concept word, unique identifier, synonyms, population supplementary concept word, anatomy supplementary concept word] | 422 |
| 18 | Leadership approach*.mp. | 168 |
| 19 | (Effective adj2 leadership).mp. [mp=title, book title, abstract, original title, name of substance word, subject heading word, floating sub-heading word, keyword heading word, organism supplementary concept word, protocol supplementary concept word, rare disease supplementary concept word, unique identifier, synonyms, population supplementary concept word, anatomy supplementary concept word] | 1108 |
| 20 | (Successful adj2 leadership).mp. [mp=title, book title, abstract, original title, name of substance word, subject heading word, floating sub-heading word, keyword heading word, organism supplementary concept word, protocol supplementary concept word, rare disease supplementary concept word, unique identifier, synonyms, population supplementary concept word, anatomy supplementary concept word] | 158 |
| 21 | Leadership style*.mp. | 1395 |
| 22 | Leadership tactic*.mp. | 6 |
| 23 | ((innovative or adaptive or transformational or situational) adj1 leadership).mp. [mp=title, book title, abstract, original title, name of substance word, subject heading word, floating sub-heading word, keyword heading word, organism supplementary concept word, protocol supplementary concept word, rare disease supplementary concept word, unique identifier, synonyms, population supplementary concept word, anatomy supplementary concept word] | 1330 |
| 24 | (Hospital* adj2 management).mp. [mp=title, book title, abstract, original title, name of substance word, subject heading word, floating sub-heading word, keyword heading word, organism supplementary concept word, protocol supplementary concept word, rare disease supplementary concept word, unique identifier, synonyms, population supplementary concept word, anatomy supplementary concept word] | 20554 |
| 25 | PHCP*.mp. | 144 |
| 26 | 15 or 16 or 17 or 18 or 19 or 20 or 21 or 22 or 23 or 24 or 25 | 144802 |
| 27 | Decision*.mp. | 599214 |
| 28 | (emergenc* adj2 decision?making).mp. [mp=title, book title, abstract, original title, name of substance word, subject heading word, floating sub-heading word, keyword heading word, organism supplementary concept word, protocol supplementary concept word, rare disease supplementary concept word, unique identifier, synonyms, population supplementary concept word, anatomy supplementary concept word] | 8 |
| 29 | Disaster* decision*.mp. | 12 |
| 30 | Cris?s decision*.mp. | 54 |
| 31 | (Cris?s adj2 decision*).mp. [mp=title, book title, abstract, original title, name of substance word, subject heading word, floating sub-heading word, keyword heading word, organism supplementary concept word, protocol supplementary concept word, rare disease supplementary concept word, unique identifier, synonyms, population supplementary concept word, anatomy supplementary concept word] | 94 |
| 32 | Health cris?s decision*.mp. | 2 |
| 33 | Medical* emergenc* decision*.mp. | 4 |
| 34 | Hospital cris?s management.mp. | 11 |
| 35 | Decision?making process*.mp. | 82 |
| 36 | Decision process*.mp. | 5112 |
| 37 | Decision?making mechanism*.mp. | 1 |
| 38 | Decision modelling.mp. | 129 |
| 39 | Medical decision?making.mp. | 41 |
| 40 | Healthcare decision?making.mp. | 9 |
| 41 | Clinical decision* process*.mp. | 144 |
| 42 | Mental process*.mp. | 14064 |
| 43 | (Health?care adj2 response).mp. [mp=title, book title, abstract, original title, name of substance word, subject heading word, floating sub-heading word, keyword heading word, organism supplementary concept word, protocol supplementary concept word, rare disease supplementary concept word, unique identifier, synonyms, population supplementary concept word, anatomy supplementary concept word] | 454 |
| 44 | (Medical adj2 preparedness).mp. [mp=title, book title, abstract, original title, name of substance word, subject heading word, floating sub-heading word, keyword heading word, organism supplementary concept word, protocol supplementary concept word, rare disease supplementary concept word, unique identifier, synonyms, population supplementary concept word, anatomy supplementary concept word] | 409 |
| 45 | 27 or 28 or 29 or 30 or 31 or 32 or 33 or 34 or 35 or 36 or 37 or 38 or 39 or 40 or 41 or 42 or 43 or 44 | 613152 |
| 46 | Infectious disease*.mp. | 159980 |
| 47 | Public health disaster*.mp. | 247 |
| 48 | cris?s in public health.mp. | 24 |
| 49 | (Disease adj2 outbreak).mp. [mp=title, book title, abstract, original title, name of substance word, subject heading word, floating sub-heading word, keyword heading word, organism supplementary concept word, protocol supplementary concept word, rare disease supplementary concept word, unique identifier, synonyms, population supplementary concept word, anatomy supplementary concept word] | 4928 |
| 50 | Pandemic*.mp. | 285292 |
| 51 | Epidemic*.mp. | 153735 |
| 52 | (Bioterrorism adj2 public health).mp. [mp=title, book title, abstract, original title, name of substance word, subject heading word, floating sub-heading word, keyword heading word, organism supplementary concept word, protocol supplementary concept word, rare disease supplementary concept word, unique identifier, synonyms, population supplementary concept word, anatomy supplementary concept word] | 53 |
| 53 | (Environment* adj1 health cris?s).mp. [mp=title, book title, abstract, original title, name of substance word, subject heading word, floating sub-heading word, keyword heading word, organism supplementary concept word, protocol supplementary concept word, rare disease supplementary concept word, unique identifier, synonyms, population supplementary concept word, anatomy supplementary concept word] | 15 |
| 54 | 46 or 47 or 48 or 49 or 50 or 51 or 52 or 53 | 561506 |
| 55 | Cris?s.mp. | 102828 |
| 56 | Emergency* response.mp. | 4222 |
| 57 | Emergency* preparedness.mp. | 2763 |
| 58 | Emergency management.mp. | 4133 |
| 59 | Disaster* response.mp. | 1913 |
| 60 | Disaster* preparedness.mp. | 2136 |
| 61 | Disaster* management.mp. | 1872 |
| 62 | Cris?s response.mp. | 501 |
| 63 | Cris?s preparedness.mp. | 75 |
| 64 | Cris?s management.mp. | 1831 |
| 65 | Emergenc* management.mp. | 4172 |
| 66 | Disaster* management.mp. | 1872 |
| 67 | (Critical adj1 care).mp. [mp=title, book title, abstract, original title, name of substance word, subject heading word, floating sub-heading word, keyword heading word, organism supplementary concept word, protocol supplementary concept word, rare disease supplementary concept word, unique identifier, synonyms, population supplementary concept word, anatomy supplementary concept word] | 90636 |
| 68 | 55 or 56 or 57 or 58 or 59 or 60 or 61 or 62 or 63 or 64 or 65 or 66 or 67 | 205801 |
| 69 | 14 and 26 and 45 and 54 and 68 | 109 |

**Updated Ovid MEDLINE search on 12^th^ December 2025**

|  | **Searches** | **Results** |  |  |  |
| --- | --- | --- | --- | --- | --- |
| 1 | Health?care.mp. or exp Healthcare Disparities/ or exp "Healthcare Failure Mode and Effect Analysis"/ | 579846 |  |  |  |
| 2 | Health?care.mp. | 579846 |  |  |  |
| 3 | Clinical.mp. | 7777350 |  |  |  |
| 4 | Medical.mp. | 2970135 |  |  |  |
| 5 | Hospital*.mp. | 2985804 |  |  |  |
| 6 | Health system*.mp. | 123148 |  |  |  |
| 7 | primary health*.mp. | 180651 |  |  |  |
| 8 | public health*.mp. | 666216 |  |  |  |
| 9 | population health*.mp. | 27546 |  |  |  |
| 10 | community health*.mp. | 140137 |  |  |  |
| 11 | (health* adj2 organi?ation*).mp. [mp=title, book title, abstract, original title, name of substance word, subject heading word, floating sub-heading word, keyword heading word, organism supplementary concept word, protocol supplementary concept word, rare disease supplementary concept word, unique identifier, synonyms, population supplementary concept word, anatomy supplementary concept word] | 252738 |  |  |  |
| 12 | patient care management.mp. | 7956 |  |  |  |
| 13 | Emergency department*.mp. | 194011 |  |  |  |
| 14 | 1 or 2 or 3 or 4 or 5 or 6 or 7 or 8 or 9 or 10 or 11 or 12 or 13 | 12574197 |  |  |  |
| 15 | Leader*.mp. | 187318 |  |  |  |
| 16 | Leadership strateg*.mp. | 406 |  |  |  |
| 17 | (Strateg* adj1 leadership).mp. [mp=title, book title, abstract, original title, name of substance word, subject heading word, floating sub-heading word, keyword heading word, organism supplementary concept word, protocol supplementary concept word, rare disease supplementary concept word, unique identifier, synonyms, population supplementary concept word, anatomy supplementary concept word] | 633 |  |  |  |
| 18 | Leadership approach*.mp. | 252 |  |  |  |
| 19 | (Effective adj2 leadership).mp. [mp=title, book title, abstract, original title, name of substance word, subject heading word, floating sub-heading word, keyword heading word, organism supplementary concept word, protocol supplementary concept word, rare disease supplementary concept word, unique identifier, synonyms, population supplementary concept word, anatomy supplementary concept word] | 1662 |  |  |  |
| 20 | (Successful adj2 leadership).mp. [mp=title, book title, abstract, original title, name of substance word, subject heading word, floating sub-heading word, keyword heading word, organism supplementary concept word, protocol supplementary concept word, rare disease supplementary concept word, unique identifier, synonyms, population supplementary concept word, anatomy supplementary concept word] | 237 |  |  |  |
| 21 | Leadership style*.mp. | 2092 |  |  |  |
| 22 | Leadership tactic*.mp. | 9 |  |  |  |
| 23 | ((innovative or adaptive or transformational or situational) adj1 leadership).mp. [mp=title, book title, abstract, original title, name of substance word, subject heading word, floating sub-heading word, keyword heading word, organism supplementary concept word, protocol supplementary concept word, rare disease supplementary concept word, unique identifier, synonyms, population supplementary concept word, anatomy supplementary concept word] | 1995 |  |  |  |
| 24 | (Hospital* adj2 management).mp. [mp=title, book title, abstract, original title, name of substance word, subject heading word, floating sub-heading word, keyword heading word, organism supplementary concept word, protocol supplementary concept word, rare disease supplementary concept word, unique identifier, synonyms, population supplementary concept word, anatomy supplementary concept word] | 30831 |  |  |  |
| 25 | PHCP*.mp. | 216 |  |  |  |
| 26 | 15 or 16 or 17 or 18 or 19 or 20 or 21 or 22 or 23 or 24 or 25 | 217203 |  |  |  |
| 27 | Decision*.mp. | 898821 |  |  |  |
| 28 | (emergenc* adj2 decision?making).mp. [mp=title, book title, abstract, original title, name of substance word, subject heading word, floating sub-heading word, keyword heading word, organism supplementary concept word, protocol supplementary concept word, rare disease supplementary concept word, unique identifier, synonyms, population supplementary concept word, anatomy supplementary concept word] | 12 |  |  |  |
| 29 | Disaster* decision*.mp. | 18 |  |  |  |
| 30 | Cris?s decision*.mp. | 81 |  |  |  |
| 31 | (Cris?s adj2 decision*).mp. [mp=title, book title, abstract, original title, name of substance word, subject heading word, floating sub-heading word, keyword heading word, organism supplementary concept word, protocol supplementary concept word, rare disease supplementary concept word, unique identifier, synonyms, population supplementary concept word, anatomy supplementary concept word] | 141 |  |  |  |
| 32 | Health cris?s decision*.mp. | 3 |  |  |  |
| 33 | Medical* emergenc* decision*.mp. | 6 |  |  |  |
| 34 | Hospital cris?s management.mp. | 16 |  |  |  |
| 35 | Decision?making process*.mp. | 123 |  |  |  |
| 36 | Decision process*.mp. | 7668 |  |  |  |
| 37 | Decision?making mechanism*.mp. | 15 |  |  |  |
| 38 | Decision modelling.mp. | 193 |  |  |  |
| 39 | Medical decision?making.mp. | 61 |  |  |  |
| 40 | Healthcare decision?making.mp. | 13 |  |  |  |
| 41 | Clinical decision* process*.mp. | 216 |  |  |  |
| 42 | Mental process*.mp. | 21096 |  |  |  |
| 43 | (Health?care adj2 response).mp. [mp=title, book title, abstract, original title, name of substance word, subject heading word, floating sub-heading word, keyword heading word, organism supplementary concept word, protocol supplementary concept word, rare disease supplementary concept word, unique identifier, synonyms, population supplementary concept word, anatomy supplementary concept word] | 681 |  |  |  |
| 44 | (Medical adj2 preparedness).mp. [mp=title, book title, abstract, original title, name of substance word, subject heading word, floating sub-heading word, keyword heading word, organism supplementary concept word, protocol supplementary concept word, rare disease supplementary concept word, unique identifier, synonyms, population supplementary concept word, anatomy supplementary concept word] | 613 |  |  |  |
| 45 | 27 or 28 or 29 or 30 or 31 or 32 or 33 or 34 or 35 or 36 or 37 or 38 or 39 or 40 or 41 or 42 or 43 or 44 | 919728 |  |  |  |
| 46 | Infectious disease*.mp. | 239970 |  |  |  |
| 47 | Public health disaster*.mp. | 370 |  |  |  |
| 48 | cris?s in public health.mp. | 36 |  |  |  |
| 49 | (Disease adj2 outbreak).mp. [mp=title, book title, abstract, original title, name of substance word, subject heading word, floating sub-heading word, keyword heading word, organism supplementary concept word, protocol supplementary concept word, rare disease supplementary concept word, unique identifier, synonyms, population supplementary concept word, anatomy supplementary concept word] | 7392 |  |  |  |
| 50 | Pandemic*.mp. | 27150 |  |  |  |
| 51 | Epidemic*.mp. | 427938 |  |  |  |
| 52 | (Bioterrorism adj2 public health).mp. [mp=title, book title, abstract, original title, name of substance word, subject heading word, floating sub-heading word, keyword heading word, organism supplementary concept word, protocol supplementary concept word, rare disease supplementary concept word, unique identifier, synonyms, population supplementary concept word, anatomy supplementary concept word] | 230602 |  |  |  |
| 53 | (Environment* adj1 health cris?s).mp. [mp=title, book title, abstract, original title, name of substance word, subject heading word, floating sub-heading word, keyword heading word, organism supplementary concept word, protocol supplementary concept word, rare disease supplementary concept word, unique identifier, synonyms, population supplementary concept word, anatomy supplementary concept word] | 79 |  |  |  |
| 54 | 46 or 47 or 48 or 49 or 50 or 51 or 52 or 53 | 22 |  |  |  |
| 55 | Cris?s.mp. | 842259 |  |  |  |
| 56 | Emergency* response.mp. | 154242 |  |  |  |
| 57 | Emergency* preparedness.mp. | 6333 |  |  |  |
| 58 | Emergency management.mp. | 4144 |  |  |  |
| 59 | Disaster* response.mp. | 6199 |  |  |  |
| 60 | Disaster* preparedness.mp. | 2869 |  |  |  |
| 61 | Disaster* management.mp. | 3204 |  |  |  |
| 62 | Cris?s response.mp. | 2808 |  |  |  |
| 63 | Cris?s preparedness.mp. | 751 |  |  |  |
| 64 | Cris?s management.mp. | 112 |  |  |  |
| 65 | Emergenc* management.mp. | 2746 |  |  |  |
| 66 | Disaster* management.mp. | 6258 |  |  |  |
| 67 | (Critical adj1 care).mp. [mp=title, book title, abstract, original title, name of substance word, subject heading word, floating sub-heading word, keyword heading word, organism supplementary concept word, protocol supplementary concept word, rare disease supplementary concept word, unique identifier, synonyms, population supplementary concept word, anatomy supplementary concept word] | 2808 |  |  |  |
| 68 | 55 or 56 or 57 or 58 or 59 or 60 or 61 or 62 or 63 or 64 or 65 or 66 or 67 | 308701 |  |  |  |
| 69 | 14 and 26 and 45 and 54 and 68 | 262 |  |  |  |

**Embase (Ovid) search on 24^th^ January 2024**

|  | **Searches** | **Results** | **Type** |
| --- | --- | --- | --- |
| 1 | (Health?care or Clinical or Medical or Hospital* or Health system* or primary health* or public health* or population health* or community health* or (health* adj2 organi?ation*) or (Health?care* adj1 organi?ation*) or patient care management or Emergency department*).mp. [mp=title, abstract, heading word, drug trade name, original title, device manufacturer, drug manufacturer, device trade name, keyword heading word, floating subheading word, candidate term word] | 17239196 | Advanced |
| 2 | (Leader* or Leadership strateg* or (Strateg* adj1 leadership) or Leadership approach* or (Effective adj2 leadership) or (Successful adj2 leadership) or Leadership style* or Leadership tactic* or ((innovative or adaptive or transformational or situational) adj1 leadership) or (Hospital* adj2 management) or PHCP*).mp. [mp=title, abstract, heading word, drug trade name, original title, device manufacturer, drug manufacturer, device trade name, keyword heading word, floating subheading word, candidate term word] | 213980 | Advanced |
| 3 | (Decision* or (emergenc* adj2 decision?making) or Disaster decision?making or Cris?s decision?making or Health?care cris?s decision* or Medical* emergenc* decision* or Hospital cris?s management or (Decision strategy* adj1 cris?s) or (Decision* process* adj1 emergenc*) or (Decision?making adj2 situation*) or Decision?making process* or Decision process* or Decision?making mechanism* or Decision modelling or Decision?making pathway* or Medical decision?making or Healthcare decision?making or Clinical decision* process* or Mental process* or (Health?care adj2 response) or (Medical adj2 preparedness)).mp. [mp=title, abstract, heading word, drug trade name, original title, device manufacturer, drug manufacturer, device trade name, keyword heading word, floating subheading word, candidate term word] | 982373 | Advanced |
| 4 | (Global health crisis* or Public health Cris?s * or (Public Health adj2 emergenc*) or Infectious disease* or Public health disaster* or cris?s in public health or (Disease adj2 outbreak) or Pandemic* or Epidemic* or (Bioterrorism adj2 public health) or (Environment* adj1 health cris?s)) )).mp. [mp=title, abstract, heading word, drug trade name, original title, device manufacturer, drug manufacturer, device trade name, keyword heading word, floating subheading word, candidate term word] | 456913 | Advanced |
| 5 | (Cris?s or Emergency* response or Emergency* preparedness or Emergency management or Disaster* response or Disaster* preparedness or Disaster* management or Cris?s response or Cris?s preparedness or Cris?s management or Emergenc* management or Disaster* management or (Critical adj1 care)).mp. [mp=title, abstract, heading word, drug trade name, original title, device manufacturer, drug manufacturer, device trade name, keyword heading word, floating subheading word, candidate term word] | 223729 | Advanced |
| 6 | 1 and 2 and 3 and 4 and 5 | 1007 | Advanced |

**Updated Embase (Ovid) search 12^th^ December 2025**

|  | **Searches** | **Results** | **Type** |
| --- | --- | --- | --- |
| 1 | (Health?care or Clinical or Medical or Hospital* or Health system* or primary health* or public health* or population health* or community health* or (health* adj2 organi?ation*) or (Health?care* adj1 organi?ation*) or patient care management or Emergency department*).mp. [mp=title, abstract, heading word, drug trade name, original title, device manufacturer, drug manufacturer, device trade name, keyword heading word, floating subheading word, candidate term word] | 17239282 | Advanced |
| 2 | (Leader* or Leadership strateg* or (Strateg* adj1 leadership) or Leadership approach* or (Effective adj2 leadership) or (Successful adj2 leadership) or Leadership style* or Leadership tactic* or ((innovative or adaptive or transformational or situational) adj1 leadership) or (Hospital* adj2 management) or PHCP*).mp. [mp=title, abstract, heading word, drug trade name, original title, device manufacturer, drug manufacturer, device trade name, keyword heading word, floating subheading word, candidate term word] | 214066 | Advanced |
| 3 | (Decision* or (emergenc* adj2 decision?making) or Disaster decision?making or Cris?s decision?making or Health?care cris?s decision* or Medical* emergenc* decision* or Hospital cris?s management or (Decision strategy* adj1 cris?s) or (Decision* process* adj1 emergenc*) or (Decision?making adj2 situation*) or Decision?making process* or Decision process* or Decision?making mechanism* or Decision modelling or Decision?making pathway* or Medical decision?making or Healthcare decision?making or Clinical decision* process* or Mental process* or (Health?care adj2 response) or (Medical adj2 preparedness)).mp. [mp=title, abstract, heading word, drug trade name, original title, device manufacturer, drug manufacturer, device trade name, keyword heading word, floating subheading word, candidate term word] | 982459 | Advanced |
| 4 | (Global health crisis* or Public health Cris?s * or (Public Health adj2 emergenc*) or Infectious disease* or Public health disaster* or cris?s in public health or (Disease adj2 outbreak) or Pandemic* or Epidemic* or (Bioterrorism adj2 public health) or (Environment* adj1 health cris?s)) )).mp. [mp=title, abstract, heading word, drug trade name, original title, device manufacturer, drug manufacturer, device trade name, keyword heading word, floating subheading word, candidate term word] | 456999 | Advanced |
| 5 | (Cris?s or Emergency* response or Emergency* preparedness or Emergency management or Disaster* response or Disaster* preparedness or Disaster* management or Cris?s response or Cris?s preparedness or Cris?s management or Emergenc* management or Disaster* management or (Critical adj1 care)).mp. [mp=title, abstract, heading word, drug trade name, original title, device manufacturer, drug manufacturer, device trade name, keyword heading word, floating subheading word, candidate term word] | 223815 | Advanced |
| 6 | 1 and 2 and 3 and 4 and 5 | 1093 | Advanced |

**PubMed Advanced search on 24^th^ January 2024**

| Search # | Query | Results |
| --- | --- | --- |
| 1 | Search: (("healthcare leader*"[Title/Abstract] OR "healthcare executive*"[Title/Abstract] OR "healthcare administrator*"[Title/Abstract] OR "medical leader*"[Title/Abstract] OR "clinical leader*"[Title/Abstract] OR "hospital administrator*"[Title/Abstract] OR "health system leader*"[Title/Abstract] OR "healthcare manager*"[Title/Abstract]) AND ((fha[Filter]) AND (1962/1/1:24/01/2024[pdat]))) OR (("leadership strategy*"[Title/Abstract] OR "Strategies in Leadership"[Title/Abstract:~0] OR "leadership approach*"[Title/Abstract] OR "Effective leadership"[Title/Abstract] OR "Successful leadership"[Title/Abstract] OR "leadership style*"[Title/Abstract] OR "Strategic leadership"[Title/Abstract] OR "leadership tactic*"[Title/Abstract] OR "Innovative leadership"[Title/Abstract] OR "Adaptive leadership"[Title/Abstract] OR "Transformational leadership"[Title/Abstract] OR "Situational leadership"[Title/Abstract] OR "leadership"[MeSH Terms] OR "experience*"[Title/Abstract] OR "reflect*"[Title/Abstract]) AND ((fha[Filter]) AND (1962/1/1:24/01/2024[pdat]))) | 1,998,134 |
| 2 | Search: ((("Emergency decision-making"[Title/Abstract] OR "Hospital crisis management"[Title/Abstract] OR "Decision-making process"[Title/Abstract] OR "Decision processes"[Title/Abstract] OR "decision making mechanism*"[Title/Abstract] OR "Decision modelling"[Title/Abstract] OR "decision making pathway*"[Title/Abstract] OR "Medical decision-making"[Title/Abstract] OR "Healthcare decision-making"[Title/Abstract] OR "Clinical decision processes"[Title/Abstract] OR ("decision making"[MeSH Terms] OR ("decision"[All Fields] AND "making"[All Fields]) OR "decision making"[All Fields])) AND ("medical subject headings"[MeSH Terms] OR ("medical"[All Fields] AND "subject"[All Fields] AND "headings"[All Fields]) OR "medical subject headings"[All Fields] OR "mesh"[All Fields])) OR "mental processes"[MeSH Terms]) AND ((fha[Filter]) AND (1962/1/1:24/01/2024[pdat])) | 982,712 |
| 3 | Search: ("hospital"[Title/Abstract] OR "community"[Title/Abstract] OR "medical care"[Title/Abstract] OR "medical industry"[Title/Abstract] OR "public health"[Title/Abstract] OR "population health"[Title/Abstract] OR "health"[MeSH Terms] OR "organizations"[MeSH Terms] OR "delivery of health care"[MeSH Terms] OR "tertiary healthcare"[MeSH Terms] OR "primary health care"[MeSH Terms] OR "patient care management"[MeSH Terms]) AND ((fha[Filter]) AND (1962/1/1:24/01/2024[pdat])) | 3,119,097 |
| 4 | Search: ("Global health crisis"[Title/Abstract] OR "Public health crisis"[Title/Abstract] OR "Public Health emergency"[Title/Abstract] OR "Infectious diseases"[Title/Abstract] OR "Public health disaster"[Title/Abstract] OR "Crisis in public health"[Title/Abstract] OR "Disease outbreak"[Title/Abstract] OR "Pandemic"[Title/Abstract] OR "Epidemic"[Title/Abstract] OR "Bioterrorism and public health"[Title/Abstract] OR "Environmental health crises) AND ((fha[Filter]) AND (1962/1/1:24/01/2024[pdat])) | 112,197 |
| 5 | Search: ("Emergency response"[Title/Abstract] OR "Emergency preparedness"[Title/Abstract] OR "Emergency management"[Title/Abstract] OR "Disaster response"[Title/Abstract] OR "Disaster preparedness"[Title/Abstract] OR "Disaster management"[Title/Abstract] OR "Healthcare emergency response"[Title/Abstract] OR "Medical emergency preparedness"[Title/Abstract] OR "Hospital emergency management"[Title/Abstract] OR "Crisis response"[Title/Abstract] OR "Crisis preparedness"[Title/Abstract] OR "Crisis management"[Title/Abstract] OR "Crisis management"[Title/Abstract] OR "Emergency management"[All Fields] OR "Disaster management"[Title/Abstract] OR "Crisis"[Title/Abstract]) AND ((fha[Filter]) AND (1962/1/1:24/01/2024[pdat])) | 84,243 |
| 1+2+3+4+5 | Search: (((("leadership strategy*"[Title/Abstract] OR "Strategies in leadership"[Title/Abstract:~0] OR "leadership approach*"[Title/Abstract] OR "Effective leadership"[Title/Abstract] OR "Successful leadership"[Title/Abstract] OR "leadership style*"[Title/Abstract] OR "Strategic leadership"[Title/Abstract] OR "leadership tactic*"[Title/Abstract] OR "Innovative leadership"[Title/Abstract] OR "Adaptive leadership"[Title/Abstract] OR "Transformational leadership"[Title/Abstract] OR "Situational leadership"[Title/Abstract] OR "leadership"[MeSH Terms] OR "experience*"[Title/Abstract] OR "reflect*"[Title/Abstract]) AND ("hasabstract"[All Fields] AND 1962/01/01:24/01/2024[Date - Publication])) OR (("healthcare leader*"[Title/Abstract] OR "healthcare executive*"[Title/Abstract] OR "healthcare administrator*"[Title/Abstract] OR "medical leader*"[Title/Abstract] OR "clinical leader*"[Title/Abstract] OR "hospital administrator*"[Title/Abstract] OR "health system leader*"[Title/Abstract] OR "healthcare manager*"[Title/Abstract]) AND ("hasabstract"[All Fields] AND 1962/01/01:24/01/2024[Date - Publication]))) AND (((("Emergency decision-making"[Title/Abstract] OR "Hospital crisis management"[Title/Abstract] OR "Decision-making process"[Title/Abstract] OR "Decision processes"[Title/Abstract] OR "decision making mechanism*"[Title/Abstract] OR "Decision modelling"[Title/Abstract] OR "decision making pathway*"[Title/Abstract] OR "Medical decision-making"[Title/Abstract] OR "Healthcare decision-making"[Title/Abstract] OR "Clinical decision processes"[Title/Abstract] OR ("decision making"[MeSH Terms] OR ("decision"[All Fields] AND "making"[All Fields]) OR "decision making"[All Fields])) AND ("medical subject headings"[MeSH Terms] OR ("medical"[All Fields] AND "subject"[All Fields] AND "headings"[All Fields]) OR "medical subject headings"[All Fields] OR "mesh"[All Fields])) OR "mental processes"[MeSH Terms]) AND ("hasabstract"[All Fields] AND 1962/01/01:24/01/2024[Date - Publication])) AND (("hospital"[Title/Abstract] OR "community"[Title/Abstract] OR "medical care"[Title/Abstract] OR "medical industry"[Title/Abstract] OR "public health"[Title/Abstract] OR "population health"[Title/Abstract] OR "health"[MeSH Terms] OR "organizations"[MeSH Terms] OR "delivery of health care"[MeSH Terms] OR "tertiary healthcare"[MeSH Terms] OR "primary health care"[MeSH Terms] OR "patient care management"[MeSH Terms]) AND ("hasabstract"[All Fields] AND 1962/01/01:24/01/2024[Date - Publication])) AND ((("Global health crisis"[Title/Abstract] OR "Public health crisis"[Title/Abstract] OR "Public Health emergency"[Title/Abstract] OR "Infectious diseases"[Title/Abstract] OR "Public health disaster"[Title/Abstract] OR "Crisis in public health"[Title/Abstract] OR "Disease outbreak"[Title/Abstract] OR "Pandemic"[Title/Abstract] OR "Epidemic"[Title/Abstract] OR "Bioterrorism and public health"[Title/Abstract] OR "Environmental health crises”[All Fields] AND 1962/01/01:24/01/2024[Date - Publication])) AND (("Emergency response"[Title/Abstract] OR "Emergency preparedness"[Title/Abstract] OR "Emergency management"[Title/Abstract] OR "Disaster response"[Title/Abstract] OR "Disaster preparedness"[Title/Abstract] OR "Disaster management"[Title/Abstract] OR "Healthcare emergency response"[Title/Abstract] OR "Medical emergency preparedness"[Title/Abstract] OR "Hospital emergency management"[Title/Abstract] OR "Crisis response"[Title/Abstract] OR "Crisis preparedness"[Title/Abstract] OR "Crisis management"[Title/Abstract] OR "Crisis management"[Title/Abstract] OR "Emergency management"[All Fields] OR "Disaster management"[Title/Abstract] OR "Crisis"[Title/Abstract]) AND ("hasabstract"[All Fields] AND 1962/01/01:24/01/2024[Date - Publication]))) AND ((fha[Filter]) AND (1962/1/1:24/01/2024[pdat])) | 517 |

**Updated PubMed Advanced search on 12^th^ December 2025**

| Search # | Query | Results |
| --- | --- | --- |
| 1 | Search: (("healthcare leader*"[Title/Abstract] OR "healthcare executive*"[Title/Abstract] OR "healthcare administrator*"[Title/Abstract] OR "medical leader*"[Title/Abstract] OR "clinical leader*"[Title/Abstract] OR "hospital administrator*"[Title/Abstract] OR "health system leader*"[Title/Abstract] OR "healthcare manager*"[Title/Abstract]) AND ((fha[Filter]) AND (1962/1/1:12/12/2025[pdat]))) OR (("leadership strategy*"[Title/Abstract] OR "Strategies in Leadership"[Title/Abstract:~0] OR "leadership approach*"[Title/Abstract] OR "Effective leadership"[Title/Abstract] OR "Successful leadership"[Title/Abstract] OR "leadership style*"[Title/Abstract] OR "Strategic leadership"[Title/Abstract] OR "leadership tactic*"[Title/Abstract] OR "Innovative leadership"[Title/Abstract] OR "Adaptive leadership"[Title/Abstract] OR "Transformational leadership"[Title/Abstract] OR "Situational leadership"[Title/Abstract] OR "leadership"[MeSH Terms] OR "experience*"[Title/Abstract] OR "reflect*"[Title/Abstract]) AND ((fha[Filter]) AND (1962/1/1:12/12/2025[pdat]))) | 2,397,760 |
| 2 | Search: ((("Emergency decision-making"[Title/Abstract] OR "Hospital crisis management"[Title/Abstract] OR "Decision-making process"[Title/Abstract] OR "Decision processes"[Title/Abstract] OR "decision making mechanism*"[Title/Abstract] OR "Decision modelling"[Title/Abstract] OR "decision making pathway*"[Title/Abstract] OR "Medical decision-making"[Title/Abstract] OR "Healthcare decision-making"[Title/Abstract] OR "Clinical decision processes"[Title/Abstract] OR ("decision making"[MeSH Terms] OR ("decision"[All Fields] AND "making"[All Fields]) OR "decision making"[All Fields])) AND ("medical subject headings"[MeSH Terms] OR ("medical"[All Fields] AND "subject"[All Fields] AND "headings"[All Fields]) OR "medical subject headings"[All Fields] OR "mesh"[All Fields])) OR "mental processes"[MeSH Terms]) AND ((fha[Filter]) AND (1962/1/1:12/12/2025[pdat])) | 1,179,254 |
| 3 | Search: ("hospital"[Title/Abstract] OR "community"[Title/Abstract] OR "medical care"[Title/Abstract] OR "medical industry"[Title/Abstract] OR "public health"[Title/Abstract] OR "population health"[Title/Abstract] OR "health"[MeSH Terms] OR "organizations"[MeSH Terms] OR "delivery of health care"[MeSH Terms] OR "tertiary healthcare"[MeSH Terms] OR "primary health care"[MeSH Terms] OR "patient care management"[MeSH Terms]) AND ((fha[Filter]) AND (1962/1/1:12/12/2025[pdat])) | 3,742,916 |
| 4 | Search: ("Global health crisis"[Title/Abstract] OR "Public health crisis"[Title/Abstract] OR "Public Health emergency"[Title/Abstract] OR "Infectious diseases"[Title/Abstract] OR "Public health disaster"[Title/Abstract] OR "Crisis in public health"[Title/Abstract] OR "Disease outbreak"[Title/Abstract] OR "Pandemic"[Title/Abstract] OR "Epidemic"[Title/Abstract] OR "Bioterrorism and public health"[Title/Abstract] OR "Environmental health crises) AND ((fha[Filter]) AND (1962/1/1:12/12/2025[pdat])) | 134,636 |
| 5 | Search: ("Emergency response"[Title/Abstract] OR "Emergency preparedness"[Title/Abstract] OR "Emergency management"[Title/Abstract] OR "Disaster response"[Title/Abstract] OR "Disaster preparedness"[Title/Abstract] OR "Disaster management"[Title/Abstract] OR "Healthcare emergency response"[Title/Abstract] OR "Medical emergency preparedness"[Title/Abstract] OR "Hospital emergency management"[Title/Abstract] OR "Crisis response"[Title/Abstract] OR "Crisis preparedness"[Title/Abstract] OR "Crisis management"[Title/Abstract] OR "Crisis management"[Title/Abstract] OR "Emergency management"[All Fields] OR "Disaster management"[Title/Abstract] OR "Crisis"[Title/Abstract]) AND ((fha[Filter]) AND (1962/1/1:12/12/2025[pdat])) | 101,091 |
| 1+2+3+4+5 | Search: (((("leadership strategy*"[Title/Abstract] OR "Strategies in leadership"[Title/Abstract:~0] OR "leadership approach*"[Title/Abstract] OR "Effective leadership"[Title/Abstract] OR "Successful leadership"[Title/Abstract] OR "leadership style*"[Title/Abstract] OR "Strategic leadership"[Title/Abstract] OR "leadership tactic*"[Title/Abstract] OR "Innovative leadership"[Title/Abstract] OR "Adaptive leadership"[Title/Abstract] OR "Transformational leadership"[Title/Abstract] OR "Situational leadership"[Title/Abstract] OR "leadership"[MeSH Terms] OR "experience*"[Title/Abstract] OR "reflect*"[Title/Abstract]) AND ("hasabstract"[All Fields] AND 1962/01/01:12/12/2025[Date - Publication])) OR (("healthcare leader*"[Title/Abstract] OR "healthcare executive*"[Title/Abstract] OR "healthcare administrator*"[Title/Abstract] OR "medical leader*"[Title/Abstract] OR "clinical leader*"[Title/Abstract] OR "hospital administrator*"[Title/Abstract] OR "health system leader*"[Title/Abstract] OR "healthcare manager*"[Title/Abstract]) AND ("hasabstract"[All Fields] AND 1962/01/01:12/12/2025[Date - Publication]))) AND (((("Emergency decision-making"[Title/Abstract] OR "Hospital crisis management"[Title/Abstract] OR "Decision-making process"[Title/Abstract] OR "Decision processes"[Title/Abstract] OR "decision making mechanism*"[Title/Abstract] OR "Decision modelling"[Title/Abstract] OR "decision making pathway*"[Title/Abstract] OR "Medical decision-making"[Title/Abstract] OR "Healthcare decision-making"[Title/Abstract] OR "Clinical decision processes"[Title/Abstract] OR ("decision making"[MeSH Terms] OR ("decision"[All Fields] AND "making"[All Fields]) OR "decision making"[All Fields])) AND ("medical subject headings"[MeSH Terms] OR ("medical"[All Fields] AND "subject"[All Fields] AND "headings"[All Fields]) OR "medical subject headings"[All Fields] OR "mesh"[All Fields])) OR "mental processes"[MeSH Terms]) AND ("hasabstract"[All Fields] AND 1962/01/01:12/12/2025[Date - Publication])) AND (("hospital"[Title/Abstract] OR "community"[Title/Abstract] OR "medical care"[Title/Abstract] OR "medical industry"[Title/Abstract] OR "public health"[Title/Abstract] OR "population health"[Title/Abstract] OR "health"[MeSH Terms] OR "organizations"[MeSH Terms] OR "delivery of health care"[MeSH Terms] OR "tertiary healthcare"[MeSH Terms] OR "primary health care"[MeSH Terms] OR "patient care management"[MeSH Terms]) AND ("hasabstract"[All Fields] AND 1962/01/01:12/12/2025[Date - Publication])) AND ((("Global health crisis"[Title/Abstract] OR "Public health crisis"[Title/Abstract] OR "Public Health emergency"[Title/Abstract] OR "Infectious diseases"[Title/Abstract] OR "Public health disaster"[Title/Abstract] OR "Crisis in public health"[Title/Abstract] OR "Disease outbreak"[Title/Abstract] OR "Pandemic"[Title/Abstract] OR "Epidemic"[Title/Abstract] OR "Bioterrorism and public health"[Title/Abstract] OR "Environmental health crises”[All Fields] AND 1962/01/01:12/12/2025[Date - Publication])) AND (("Emergency response"[Title/Abstract] OR "Emergency preparedness"[Title/Abstract] OR "Emergency management"[Title/Abstract] OR "Disaster response"[Title/Abstract] OR "Disaster preparedness"[Title/Abstract] OR "Disaster management"[Title/Abstract] OR "Healthcare emergency response"[Title/Abstract] OR "Medical emergency preparedness"[Title/Abstract] OR "Hospital emergency management"[Title/Abstract] OR "Crisis response"[Title/Abstract] OR "Crisis preparedness"[Title/Abstract] OR "Crisis management"[Title/Abstract] OR "Crisis management"[Title/Abstract] OR "Emergency management"[All Fields] OR "Disaster management"[Title/Abstract] OR "Crisis"[Title/Abstract]) AND ("hasabstract"[All Fields] AND 1962/01/01:12/12/2025[Date - Publication]))) AND ((fha[Filter]) AND (1962/1/1:12/12/2025[pdat])) | 534 |

**ProQuest search 24/01/2024**

| Query | Results |
| --- | --- |
| abstract(((Healthcare leader) OR (Healthcare executive) OR (Healthcare administrator) OR (Medical leader) OR (Clinical leader) OR (Hospital administrator) OR (Health system leader) OR (Healthcare manager))) AND abstract(((Leadership strategy) OR (Strategies in leadership) OR (Leadership approach) OR (Effective leadership) OR (Successful leadership) OR (Leadership style) OR (Strategic leadership) (Leadership tactic) OR (Innovative leadership) OR (Adaptive leadership) OR (Transformational leadership) OR (Situational leadership) OR (leadership) OR (experience) OR (reflect))) AND abstract(((Emergency decision making) OR (Disaster decision making) OR (Healthcare crisis decision) OR (Medical emergency decision) OR (Hospital crisis management) OR (Decision strategies in crises) OR (Decision processes during emergency) OR (Decision-making in critical situations) OR (Decision-making process) OR (Decision processes) OR (Decision-making mechanism) OR (Decision modelling) OR (Decision-making pathway) OR (Medical decision making) OR (Healthcare decision making) OR (Clinical decision processes) OR (decision making) OR (mental processes))) AND abstract(((hospital) OR (community) OR (medical care) OR (medical industry) OR (public health) OR (population health) OR (health) (organizations) OR (delivery of health care) OR (tertiary healthcare) OR (primary health care) OR (patient care management))) AND abstract (((Global health crisis) OR (Public health crisis) OR (Public Health emergency) OR (Infectious diseases) OR (Public health disaster) OR (Crisis in public health) OR (Disease outbreak) OR (Pandemic) OR (Epidemic) OR (Bioterrorism and public health) OR (Environmental health crises)))  AND abstract (((Emergency response) OR (Emergency preparedness) OR (Emergency management) OR (Disaster response) OR (Disaster preparedness) OR (Disaster management) OR (Healthcare emergency response) OR (Medical emergency preparedness) OR (Hospital emergency management) OR (Crisis response) OR (Crisis preparedness) OR (Crisis management) OR (Crisis management) OR (Emergency management) OR (Disaster management) OR (Crisis) OR (critical care))) | 246 |

**Updated ProQuest search on 12^th^ December 2025**

| Query | Results |
| --- | --- |
| abstract(((Healthcare leader) OR (Healthcare executive) OR (Healthcare administrator) OR (Medical leader) OR (Clinical leader) OR (Hospital administrator) OR (Health system leader) OR (Healthcare manager))) AND abstract(((Leadership strategy) OR (Strategies in leadership) OR (Leadership approach) OR (Effective leadership) OR (Successful leadership) OR (Leadership style) OR (Strategic leadership) (Leadership tactic) OR (Innovative leadership) OR (Adaptive leadership) OR (Transformational leadership) OR (Situational leadership) OR (leadership) OR (experience) OR (reflect))) AND abstract(((Emergency decision making) OR (Disaster decision making) OR (Healthcare crisis decision) OR (Medical emergency decision) OR (Hospital crisis management) OR (Decision strategies in crises) OR (Decision processes during emergency) OR (Decision-making in critical situations) OR (Decision-making process) OR (Decision processes) OR (Decision-making mechanism) OR (Decision modelling) OR (Decision-making pathway) OR (Medical decision making) OR (Healthcare decision making) OR (Clinical decision processes) OR (decision making) OR (mental processes))) AND abstract(((hospital) OR (community) OR (medical care) OR (medical industry) OR (public health) OR (population health) OR (health) (organizations) OR (delivery of health care) OR (tertiary healthcare) OR (primary health care) OR (patient care management))) AND abstract (((Global health crisis) OR (Public health crisis) OR (Public Health emergency) OR (Infectious diseases) OR (Public health disaster) OR (Crisis in public health) OR (Disease outbreak) OR (Pandemic) OR (Epidemic) OR (Bioterrorism and public health) OR (Environmental health crises)))  AND abstract (((Emergency response) OR (Emergency preparedness) OR (Emergency management) OR (Disaster response) OR (Disaster preparedness) OR (Disaster management) OR (Healthcare emergency response) OR (Medical emergency preparedness) OR (Hospital emergency management) OR (Crisis response) OR (Crisis preparedness) OR (Crisis management) OR (Crisis management) OR (Emergency management) OR (Disaster management) OR (Crisis) OR (critical care))) | 268 |

**Scopus search on 24^th^ January 2024**

| Query | Results |
| --- | --- |
| ( TITLE-ABS-KEY ( ( ( healthcare AND leader ) OR ( healthcare AND executive ) OR ( healthcare AND administrator ) OR ( medical AND leader ) OR ( clinical AND leader ) OR ( hospital AND administrator ) OR ( health AND system AND leader ) OR ( healthcare AND manager ) ) ) AND TITLE-ABS-KEY ( ( ( leadership AND strategy ) OR ( strategies AND in AND leadership ) OR ( leadership AND approach ) OR ( effective AND leadership ) OR ( successful AND leadership ) OR ( leadership AND style ) OR ( strategic AND leadership ) ( leadership AND tactic ) OR ( innovative AND leadership ) OR ( adaptive AND leadership ) OR ( transformational AND leadership ) OR ( situational AND leadership ) OR ( leadership ) OR ( experience ) OR ( reflect ) ) ) AND TITLE-ABS-KEY ( ( ( emergency AND decision AND making ) OR ( disaster AND decision AND making ) OR ( healthcare AND crisis AND decision ) OR ( medical AND emergency AND decision ) OR ( hospital AND crisis AND management ) OR ( decision AND strategies AND in AND crises ) OR ( decision AND processes AND during AND emergency ) OR ( decision-making AND in AND critical AND situations ) OR ( decision-making AND process ) OR ( decision AND processes ) OR ( decision-making AND mechanism ) OR ( decision AND modelling ) OR ( decision-making AND pathway ) OR ( medical AND decision AND making ) OR ( healthcare AND decision AND making ) OR ( clinical AND decision AND processes ) OR ( decision AND making ) OR ( mental AND processes ) ) ) AND TITLE-ABS-KEY ( ( ( hospital ) OR ( community ) OR ( medical AND care ) OR ( medical AND industry ) OR ( public AND health ) OR ( population AND health ) OR ( health ) ( organizations ) OR ( delivery AND of AND health AND care ) OR ( tertiary AND healthcare ) OR ( primary AND health AND care ) OR ( patient AND care AND management ) ) ) AND TITLE-ABS-KEY (((Global AND health AND crisis) OR (Public AND health AND crisis) OR (Public health AND emergency) OR (Infectious AND diseases) OR (Public AND health AND disaster) OR (Crisis AND in AND public AND health) OR (Disease AND outbreak) OR (Pandemic) OR (Epidemic) OR (Bioterrorism AND public AND health) OR (Environmental AND health AND crises))) AND TITLE-ABS-KEY ( ( ( emergency AND response ) OR ( emergency AND preparedness ) OR ( emergency AND management ) OR ( disaster AND response ) OR ( disaster AND preparedness ) OR ( disaster AND management ) OR ( healthcare AND emergency AND response ) OR ( medical AND emergency AND preparedness ) OR ( hospital AND emergency AND management ) OR ( crisis AND response ) OR ( crisis AND preparedness ) OR ( crisis AND management ) OR ( crisis AND management ) OR ( emergency AND management ) OR ( disaster AND management ) OR ( crisis ) OR ( critical AND care ) ) ) ) AND PUBYEAR > 1962 AND PUBYEAR < 2024 | 557 |

**Updated Scopus search on 12^th^ December 2025**

| Query | Results |
| --- | --- |
| ( TITLE-ABS-KEY ( ( ( healthcare AND leader ) OR ( healthcare AND executive ) OR ( healthcare AND administrator ) OR ( medical AND leader ) OR ( clinical AND leader ) OR ( hospital AND administrator ) OR ( health AND system AND leader ) OR ( healthcare AND manager ) ) ) AND TITLE-ABS-KEY ( ( ( leadership AND strategy ) OR ( strategies AND in AND leadership ) OR ( leadership AND approach ) OR ( effective AND leadership ) OR ( successful AND leadership ) OR ( leadership AND style ) OR ( strategic AND leadership ) ( leadership AND tactic ) OR ( innovative AND leadership ) OR ( adaptive AND leadership ) OR ( transformational AND leadership ) OR ( situational AND leadership ) OR ( leadership ) OR ( experience ) OR ( reflect ) ) ) AND TITLE-ABS-KEY ( ( ( emergency AND decision AND making ) OR ( disaster AND decision AND making ) OR ( healthcare AND crisis AND decision ) OR ( medical AND emergency AND decision ) OR ( hospital AND crisis AND management ) OR ( decision AND strategies AND in AND crises ) OR ( decision AND processes AND during AND emergency ) OR ( decision-making AND in AND critical AND situations ) OR ( decision-making AND process ) OR ( decision AND processes ) OR ( decision-making AND mechanism ) OR ( decision AND modelling ) OR ( decision-making AND pathway ) OR ( medical AND decision AND making ) OR ( healthcare AND decision AND making ) OR ( clinical AND decision AND processes ) OR ( decision AND making ) OR ( mental AND processes ) ) ) AND TITLE-ABS-KEY ( ( ( hospital ) OR ( community ) OR ( medical AND care ) OR ( medical AND industry ) OR ( public AND health ) OR ( population AND health ) OR ( health ) ( organizations ) OR ( delivery AND of AND health AND care ) OR ( tertiary AND healthcare ) OR ( primary AND health AND care ) OR ( patient AND care AND management ) ) ) AND TITLE-ABS-KEY (((Global AND health AND crisis) OR (Public AND health AND crisis) OR (Public health AND emergency) OR (Infectious AND diseases) OR (Public AND health AND disaster) OR (Crisis AND in AND public AND health) OR (Disease AND outbreak) OR (Pandemic) OR (Epidemic) OR (Bioterrorism AND public AND health) OR (Environmental AND health AND crises))) AND TITLE-ABS-KEY ( ( ( emergency AND response ) OR ( emergency AND preparedness ) OR ( emergency AND management ) OR ( disaster AND response ) OR ( disaster AND preparedness ) OR ( disaster AND management ) OR ( healthcare AND emergency AND response ) OR ( medical AND emergency AND preparedness ) OR ( hospital AND emergency AND management ) OR ( crisis AND response ) OR ( crisis AND preparedness ) OR ( crisis AND management ) OR ( crisis AND management ) OR ( emergency AND management ) OR ( disaster AND management ) OR ( crisis ) OR ( critical AND care ) ) ) ) AND PUBYEAR > 1962 AND PUBYEAR < 2025 | 633 |

**EBSCOhost (**Business Source Complete**) search on 24^th^ January 2024**

| **Interface/** EBSCOhost Research Databases / **Search Screen/** Advanced Search **Database/** Business Source Complete | Results |
| --- | --- |
| AB ( ((Healthcare leader) OR (Healthcare executive) OR (Healthcare administrator) OR (Medical leader) OR (Clinical leader) OR (Hospital administrator) OR (Health system leader) OR (Healthcare manager)) ) AND AB ( ((Leadership strategy) OR (Strategies in leadership) OR (Leadership approach) OR (Effective leadership) OR (Successful leadership) OR (Leadership style) OR (Strategic leadership) (Leadership tactic) OR (Innovative leadership) OR (Adaptive leadership) OR (Transformational leadership) OR (Situational leadership) OR (leadership) OR (experience) OR (reflect)) ) AND AB ( ((Emergency decision making) OR (Disaster decision making) OR (Healthcare crisis decision) OR (Medical emergency decision) OR (Hospital crisis management) OR (Decision strategies in crises) OR (Decision processes during emergency) OR (Decision-making in critical situations) OR (Decision-making process) OR (Decision processes) OR (Decision-making mechanism) OR (Decision modelling) OR (Decision-making pathway) OR (Medical decision making) OR (Healthcare decision making) OR (Clinical decision processes) OR ( decision making) OR ( mental processes)) ) AND AB ( ((hospital) OR (community) OR (medical care) OR (medical industry) OR (public health) OR (population health) OR (health) (organizations) OR ( delivery of health care) OR ( tertiary healthcare) OR ( primary health care) OR ( patient care management)) ) AND AB ( ((Global health crisis) OR (Public health crisis) OR (Public Health emergency) OR (Infectious diseases) OR (Public health disaster) OR (Crisis in public health) OR (Disease outbreak) OR (Pandemic) OR (Epidemic) OR (Bioterrorism and public health) OR (Environmental health crises)) ) AND AB ( ((Emergency response) OR (Emergency preparedness) OR (Emergency management) OR (Disaster response) OR (Disaster preparedness) OR (Disaster management) OR (Healthcare emergency response) OR (Medical emergency preparedness) OR (Hospital emergency management) OR (Crisis response) OR (Crisis preparedness) OR (Crisis management) OR (Crisis management) OR (Emergency management) OR (Disaster management) OR (Crisis) OR ( critical care)) ) | 11 |

**Updated EBSCOhost (**Business Source Complete**) search on 12^th^ December 2025**

| **Interface/** EBSCOhost Research Databases / **Search Screen/** Advanced Search **Database/** Business Source Complete | Results |
| --- | --- |
| AB ( ((Healthcare leader) OR (Healthcare executive) OR (Healthcare administrator) OR (Medical leader) OR (Clinical leader) OR (Hospital administrator) OR (Health system leader) OR (Healthcare manager)) ) AND AB ( ((Leadership strategy) OR (Strategies in leadership) OR (Leadership approach) OR (Effective leadership) OR (Successful leadership) OR (Leadership style) OR (Strategic leadership) (Leadership tactic) OR (Innovative leadership) OR (Adaptive leadership) OR (Transformational leadership) OR (Situational leadership) OR (leadership) OR (experience) OR (reflect)) ) AND AB ( ((Emergency decision making) OR (Disaster decision making) OR (Healthcare crisis decision) OR (Medical emergency decision) OR (Hospital crisis management) OR (Decision strategies in crises) OR (Decision processes during emergency) OR (Decision-making in critical situations) OR (Decision-making process) OR (Decision processes) OR (Decision-making mechanism) OR (Decision modelling) OR (Decision-making pathway) OR (Medical decision making) OR (Healthcare decision making) OR (Clinical decision processes) OR ( decision making) OR ( mental processes)) ) AND AB ( ((hospital) OR (community) OR (medical care) OR (medical industry) OR (public health) OR (population health) OR (health) (organizations) OR ( delivery of health care) OR ( tertiary healthcare) OR ( primary health care) OR ( patient care management)) ) AND AB ( ((Global health crisis) OR (Public health crisis) OR (Public Health emergency) OR (Infectious diseases) OR (Public health disaster) OR (Crisis in public health) OR (Disease outbreak) OR (Pandemic) OR (Epidemic) OR (Bioterrorism and public health) OR (Environmental health crises)) ) AND AB ( ((Emergency response) OR (Emergency preparedness) OR (Emergency management) OR (Disaster response) OR (Disaster preparedness) OR (Disaster management) OR (Healthcare emergency response) OR (Medical emergency preparedness) OR (Hospital emergency management) OR (Crisis response) OR (Crisis preparedness) OR (Crisis management) OR (Crisis management) OR (Emergency management) OR (Disaster management) OR (Crisis) OR ( critical care)) ) | 19 |

# **Supplementary File 3: Critical appraisal of included studies**.

***A-Qualitative research checklist:***

| **Included studies** | **Q1** | **Q2** | **Q3** | **Q4** | **Q5** | **Q6** | **Q7** | **Q8** | **Q9** | **Q10** | **Total** |
| --- | --- | --- | --- | --- | --- | --- | --- | --- | --- | --- | --- |
| Edwards (2024) | Y | Y | Y | Y | Y | Y | N | Y | Y | Y | 9/10 |
| Johnson (2023) | Y | Y | Y | Y | Y | Y | N | Y | Y | Y | 9/10 |
| Johnson-Cornet (2023) | Y | Y | Y | Y | Y | Y | Y | Y | Y | Y | 10/10 |
| Barishansky (2022) | Y | Y | Y | Y | Y | N | N | Y | N | Y | 7/10 |
| Carbone (2023) | Y | Y | Y | Y | Y | Y | N | Y | Y | Y | 9/10 |
| Katapally (2023) | Y | Y | Y | Y | Y | Y | N | Y | Y | Y | 9/10 |
| Khalid (2023) | Y | Y | Y | Y | Y | N | N | Y | Y | Y | 8/10 |
| Skagerström (2023) | Y | Y | Y | Y | Y | Y | N | Y | Y | Y | 9/10 |
| Pettersson (2023) | Y | Y | Y | Y | Y | N | N | Y | N | Y | 7/10 |
| Bhat (2023) | Y | Y | Y | Y | Y | N | Y | Y | Y | Y | 9/10 |
| Bastani (2022) | Y | Y | Y | Y | Y | Y | N | Y | U | Y | 8/10 |
| Clyne (2023) | Y | Y | Y | Y | Y | Y | N | Y | Y | Y | 9/10 |
| Romiti (2025) | Y | Y | Y | Y | Y | Y | N | Y | Y | Y | 9/10 |
| Phillips (2023) | Y | Y | Y | Y | Y | Y | N | U | Y | Y | 8/10 |
| Doran (2025) | Y | Y | Y | Y | Y | Y | N | Y | Y | Y | 9/10 |
| Fenton (2021) | Y | Y | Y | Y | Y | N | N | Y | Y | Y | 9/10 |
| Tseng (2005) | Y | Y | Y | Y | Y | N | N | Y | N | Y | 7/10 |
| Thomas (2009) | Y | Y | Y | Y | Y | N | N | Y | Y | Y | 8/10 |

*N, No; U, Unclear; Y, Yes*; *NA, Not Applicable*.

*Criteria for the critical appraisal of qualitative evidence:*

*Q1 = Is there congruity between the stated philosophical perspective and the research methodology?*

*Q2 = Is there congruity between the research methodology and the research question or objectives?*

*Q3 = Is there congruity between the research methodology and the methods used to collect data?*

*Q4 = Is there congruity between the research methodology and the representation and analysis of data?*

*Q5 = Is there congruity between the research methodology and the interpretation of results?*

*Q6 = Is there a statement locating the researcher culturally or theoretically?*

*Q7 = Is the influence of the researcher on the research, and vice versa, addressed?*

*Q8 = Are participants, and their voices, adequately represented?*

*Q9 = Is the research ethical according to current criteria or, for recent studies, and is there evidence of ethical approval by an appropriate body?*

*Q10: Do the conclusions drawn in the research report flow from the analysis or interpretation of the data?*

***B- Narrative Quality Appraisal (Narrative Checklist) for narrative accounts (field reports and case studies).***

| ***Study*** | ***Q1*** | ***Q2*** | ***Q3*** | ***Q4*** | ***Q5*** | ***Q6*** | **Total** |
| --- | --- | --- | --- | --- | --- | --- | --- |
| *Guidolin (2022)* | *Y* | *Y* | *Y* | *Y* | *Y* | *U* | *5/6* |
| *Rimstad (2015)* | *Y* | *Y* | *Y* | *Y* | *Y* | *Y* | *6/6* |
| *Aragón (2021)* | *Y* | *Y* | *Y* | *Y* | *Y* | *Y* | *6/6* |

*Y: Yes. N: No. U: Unclear. NA: Not applicable.*

*1: Is the generator of the narrative a credible or appropriate source?*

*2: Is the relationship between the text and its context explained? (where, when, who with, how).*

*3: Does the narrative present the events using a logical sequence so the reader or listener can understand how it unfolds?*

*4: Do you, as a reader or listener of the narrative, arrive at similar conclusions to those drawn by the narrator?*

*5: Do the conclusions flow from the narrative account? 6: Do you consider this account to be a narrative?*

# Supplementary File 4: Data extracted

| **Author /Publication Year** | **Country** | **Crisis Type /Year** | **Study type** | **Decision-maker positions** | **Aims** | **Key findings** | **Authors’ conclusions** | **Limitations** |
| --- | --- | --- | --- | --- | --- | --- | --- | --- |
| Edwards (2024) ^1^ | USA | COVID-19/2020 | Qualitative | Clinical leaders | Explore primary care leaders’ experiences and strategies during COVID‑19. | Early: rapid change and constant decisions; team purpose buffered stress.  Later: layered crises (fatigue/burnout) strained relationships.  Key strategies: presence, over‑communication, relationship‑building, adaptive decision‑making. | Relational and adaptive leadership supports teams and organisational resilience during prolonged crisis and burnout. | Small sample; recruitment via authors’ networks (selection bias).  Limited diversity of leaders/settings; reflexivity noted. |
| Johnson (2023) ^2^ | USA | COVID-19/2020 | Qualitative | Organisational decision‐makers | Examine organisational decision-making in North Carolina during COVID‑19. | Decisions shaped by scientific authorities, peer decisions, and prior crisis experience.  Decentralisation and auxiliary bodies helped manage complex inputs.  Underrepresentation of racial/ethnic minority communities in the sample. | Decision effectiveness depended on system “fit” and adaptability.  Alignment and collaboration across local organisations strengthened the response. | Timing effects not controlled; interviews across a rapidly evolving period; recall bias.  Metro/White skew limits generalisability; early‑pandemic window only. |
| Johnson-Cornet (2023) ^3^ | USA | COVID-19/2020 | Qualitative | CEOs | Identify how non‑profit healthcare CEOs make ethical decisions and avoid ethical scandals. | Themes: promoting values, fostering ethical culture and embedding ethical decisions at all levels.  Encouraged diverse worldviews and safe expression of ethical concerns.  Policies and supportive climate reduced unintended ethical consequences. | CEO strategies can reduce ethical failures and strengthen integrity and social responsibility during crises. | Small, single‑region CEO sample; transferability limited.  Only CEOs included; sector‑specific context. |
| Barishansky (2022) ^4^ | USA | COVID-19/2020 | Qualitative | State Health Officials | Understand State Health Officials’ (SHOs) DM during COVID‑19 and identify tool/training needs. | No dedicated decision tool; reliance on consensus was challenging but not avoidable.  COVID‑19 is viewed as the most complex emergency experienced.  State–local interaction and political/media dynamics strongly influenced decisions. | Structured, stepwise decision tools and workforce training are needed to support SHOs during public health emergencies. | SHO‑only perspectives; geographic overlap limits representativeness.  Qualitative interpretation and researcher presence may introduce bias. |
| Aragón (2021) ^5^ | USA | COVID-19/2020 | Field Report | Local health officers | Describe Bay Area shelter‑in‑place (SIP) decisions and advocate for decision intelligence in crisis management. | First regional SIP orders issued amid rising cases; high uncertainty about effectiveness.  Decision intelligence framed trade‑offs; consequence/strategy tables supported alternatives assessment.  Hospitals were not overwhelmed, though preparedness gaps were acknowledged. | Rapid action under uncertainty is required; decision quality should be judged by the process at the time.  Ongoing decision intelligence training recommended. | Limited knowledge of transmission and preparedness at decision time.  Outcomes alone are insufficient to evaluate decision quality. |
| Carbone (2023) ^6^ | Canada | COVID-19/2020 | Qualitative | Residents, care partners, health professionals | Examine long‑term care (LTC) transition decision-making during COVID‑19 in Ontario. | Seven factors shaped transition decisions (e.g., institutional priorities, resources, beliefs).  Professionals often dominated decisions; pandemic increased care‑partner involvement.  Emotions and resources influenced crisis appraisal and response decisions’ options. | Crisis reshaped transition decisions; decision supports/aids are needed for stakeholders.  LTC conditions drive ongoing transitions, implying policy relevance. | English‑language restriction and topic complexity may omit evidence.  Limited equity‑group perspectives; minimal patient/family involvement in factor identification. |
| Katapally (2023) ^7^ | Canada | COVID-19/2020 | Qualitative | Scientist; Advisory Council | Develop digital health dashboards using citizen data to support rapid, ethical public health decision-making. | Dashboards convert citizen data into real‑time evidence and analytics.  Enable direct communication between decision‑makers and citizens for risk management and decision-making.  Support decentralisation and data sovereignty but depend on citizen uptake. | Citizen‑driven dashboards can strengthen rapid responses and equity‑oriented systems integration. | Digital divides affect participation; ongoing security risks.  Real‑world validation needed; ethics exemption may raise concerns. |
| Khalid (2023) ^8^ | Canada | COVID-19/2020 | Mixed method | Decision-makers involved in healthcare and emergencies involving humans | Identify decision‑makers’ preferences for rapid evidence summaries in health/humanitarian crises and develop a template. | Referred concise bullet points/infographics with actionable messages.  Time pressure and access constraints shaped evidence use in the decision.  Social‑media‑friendly formats improved dissemination. | Plain‑language rapid summaries support real‑time decisions; templates should be tailored and include critical appraisal. | Non‑users are less familiar with websites; limited comparisons.  Internet constraints; topic categorisation difficulties; limited time to engage with longer documents. |
| Guidolin (2022) ^9^ | Canada | COVID-19/2020 | Observational | Interprofessional team | Develop an ethical tool to guide fair resource allocation and clinical ramp‑down during COVID‑19. | Stepwise ethical tool developed using Accountability for Reasonableness (A4R).  Uniform first‑wave ramp‑down caused inefficiency; the second wave required smarter prioritisation to decide ethically.  Equity risks persisted despite stakeholder input. | Ethical allocation tools can improve fairness under scarcity, but implementation challenges (e.g., publicity and equity risks) remain. | Program‑focused tool may miss individual patient needs.  Access inequities may exclude patients; the tool is not widely publicised. |
| Skagerström (2023) ^10^ | Sweden | COVID-19/2020 | Qualitative | Managers in primary health | Explore primary healthcare managers’ experiences of decision-making during COVID‑19. | Managers felt lonely in decision-making and stretched to limits, yet proud/resilient.  Increased demands and reduced control worsened psychosocial conditions.  Effort–reward imbalance heightened stress risk. | Leadership support systems are needed; humane and just leadership may strengthen future crisis response. | Volunteer bias; interview guide not manager-specific.  May under-capture those under the greatest strain and contextual complexity. |
| Pettersson (2023) ^11^ | Sweden | COVID-19/2020 | Qualitative | Medical command and control teams | Describe challenges in the USA burn care infrastructure and crisis management during COVID‑19. | Command teams described episodes shaped by organisation, adaptation, shared picture, assumptions, and analysis.  Burn care capacity is limited and vulnerable to surge/mass casualty events.  Observation wards improved throughput and reduced unnecessary admissions. | Resilient, adaptive systems and clearer integration of burn care into emergency plans can improve crisis care. | Resource constraints and coordination gaps across partners.  Local plans and literature scope may omit aspects of burn response. |
| Bhat (2023) ^12^ | India | COVID-19/2020 | Reflective analysis | Public health experts | Reflect on public leadership during crises and lessons from COVID‑19. | Crisis required rapid decisions, clear communication, and innovation.  Public health leaders faced political roles and complex social/ economic pressures, and only reliable data can improve their decisions.  Leadership responses influenced organisational survival. | Future readiness requires adaptable governance, technical competence, and empathetic communication. | Public health professionals often lacked political experience/ training.  Limited participation/visibility; lessons still evolving with changing crisis conditions. |
| Bastani (2022)^13^ | Iran | COVID-19/2020 | Qualitative | Senior health system managers | Examine Knowledge Translation Exchange (KTE) and evidence‑informed policymaking among Iranian decision‑makers during COVID‑19. | Ten themes on evidence use; knowledge brokers and KTE tools were important.  Barriers included negative beliefs, cultural obstacles, and limited time.  Research outputs were often underused in government decisions. | Purposive KTE strategies and activated knowledge brokers can strengthen evidence use during urgent crises. | Some interviews by phone; limited participant time reduced depth.  Context‑specific qualitative findings require cautious transfer. |
| Clyne (2023) ^14^ | Ireland | COVID-19/2020 | Qualitative | Policy makers and front-line clinicians | Explore experiences of producing/using rapid evidence products for COVID‑19 decision-making. | Rapid evidence products were highly valued for national decision-making.  Quick decisions are needed to address the crisis.  Trust and credibility were crucial; communication/website issues hindered use.  High workload requires skilled, adaptable teams. | Rapid evidence synthesis is vital for preparedness; investment in capacity, planning, and relationships is needed. | Limited provider/user pool; some authors were participants (potential bias).  No patient representatives; limited cross‑sector perspectives; workload unsustainable. |
| Romiti (2025) ^15^ | Italy | COVID-19/2020 | Qualitative | CEOs of local health authorities | Examine how crises shape strategic‑level decision-making processes in healthcare organisations. | Crisis can centralise decision processes and broaden actor involvement.  Politicisation varied with CEO stakeholder collaborations.  Reliance on CEO perceptions and decisions suggests adapting to wider stakeholder views in crisis. | Reflecting on crisis decision processes can support transition to a post‑crisis “new normal” and improve implementation governance. | Limitations not explicitly stated; implied single‑context focus and CEO‑centric perspective. |
| Phillips (2023) ^16^ | Norway | COVID-19/2020 | Case | Local authority representatives | Identify decision bottlenecks in Norway’s COVID‑19 vaccine rollout and how central authorities can support local decision-making. | Multiple bottlenecks emerged; some could be reduced by adjusting external constraints.  Coordination and shared situational awareness could support distributed improvisation.  Important roles existed beyond initial prioritisation criteria. | Central support can ease local bottlenecks through coordination and information sharing while maintaining trust and autonomy. | Restricted participant access, limited system view; few authority perspectives.  Bottleneck identification and deeper dynamics hard to capture; single‑coder approach. |
| Doran (2025) ^17^ | United Kingdom | COVID-19/2020 | Mixed method | Senior National Health Service decision-makers | Assess how the UK National Health Service (NHS) decision protocols adapted from traditional to emergency processes during COVID‑19. | Protocols shifted for speed while retaining core values (welfare, equity).  Urgency modified principles; need better quality/usability of effectiveness data.  Organisational values guided decisions but required realignment in crisis. | Crisis decision-making needs timely, clear processes and transparent communication about temporary procedural changes. | Senior‑only focus; online survey snapshot limits representativeness.  Limited frontline/mid‑level views; scalability beyond crisis unclear. |
| Fenton (2021) ^18^ | New Zealand | Ebola/2014–2016 | Qualitative | Public health emergencies decision-makers | Analyse how politicisation, urgency, uncertainty, and fear shape public health emergency decision-making. | Four conditions strongly influenced crisis decisions and research conduct.  Biomedical focus can crowd out infrastructure/system solutions; equity concerns.  Urgency and fear complicate consent and ethical reasoning. | Balance rapid biomedical action with long‑term equity and system strengthening; reassess research priorities under crisis pressures. | Blueprints may overlook structural causes (e.g., poverty, instability).  Crisis visibility can overshadow underlying drivers; moral uncertainties in trial ethics. |
| Tseng (2005) ^19^ | Taiwan | COVID/2002–2004 | Case | Nursing directors | Identify factors enabling a Taipei hospital to manage SARS-CoV2 effectively. | Five survival factors: timing, decision-making, implementation, communication, and trust.  Successful management despite a high‑risk setting; training and supplies supported unity.  Administrative focus due to limited medical countermeasures at the time. | Early action, trust, communication, and thorough implementation underpin effective crisis management. | No direct observation; nurse‑only interviews limited perspectives.  Staff fear and broader views were not deeply explored. |
| Thomas (2009) ^20^ | USA | Hurricanes/1999, 2002 Influenza/2003 | Case | Epidemiology section experts | Identify ethical issues faced by public health responders and recommend training/ process improvements. | Twenty‑four ethical issues across emergencies; communication with vulnerable groups most common.  Responders sensed dilemmas but lacked formal ethical frameworks.  Community input important; practical steps proposed. | Integrating public health ethics training and structured processes can strengthen ethical decision-making in emergencies. | Limited ethics training constrained issue identification.  Some barriers and cultural considerations were underexplored. |
| Rimstad (2015) ^21^ | Norway | Terrorist bombing/2011 | Observational | Ambulance commander | Explore critical decisions during a terrorist mass‑casualty incident and how uncertainty/ time pressure shape command decisions. | Most critical decisions occurred in the first 30 minutes under high uncertainty.  Situation assessment was central and shaped by experience/knowledge.  Uncertainty peaked after early decisions, requiring later adjustment. | Commanders need strong early situation assessment and rapid decision capacity, with flexibility as uncertainty changes. | Recall bias; radio logs miss informal communication.  Descriptive focus on what decisions were made (less on how/quality); staging framework not standardised. |

# **Supplementary File 5: Meta‑ethnographic analysis of decision‑making (DM) models, presented as a translation matrix with reciprocal and** refutational notes

| Reference | First-order constructs | Second-order constructs | Translation notes (reciprocal/refutational) | Third-order construct (DM model) |
| --- | --- | --- | --- | --- |
| Edwards (2024) | Rapid change; reassessing command-and-control; balancing directive and participatory approaches. | Adapting to change; adjusting to needs; empowerment with direction. | Reciprocal: aligns with decentralisation/adaptation as flexible authority. | Adaptive: iterative adjustment; balance control/participation. |
| Johnson (2023) | Conflict management; decentralised and rapid DM. | Adapting to conflict; local needs; crisis dynamics. | Reciprocal: complements Edwards; conflict triggers decentralisation. | Adaptive: flexible decentralised response. |
| Johnson-Cornet (2023) | Supporting ethical values; promoting ethical mindset. | Ethical approach foregrounded. | Reciprocal: aligns with structured ethical models. | Ethical: values-driven organisational practice. |
| Barishansky (2022) | No formal tool; consensus-based process. | Consensus avoids unilateral risk. | Refutational: contrasts rapid/centralised approaches. | Consensus: legitimacy via collective agreement. |
| Aragón (2021) | Legal authority; decision intelligence. | Risk/outcome evaluation. | Reciprocal: overlaps evidence-informed reasoning. | Crisis: urgent decisions under uncertainty. |
| Carbone (2023) | Multiple influencing factors; transition decisions. | Adapting to guidance; adapting to change. | Reciprocal: aligns with adaptive models in evolving contexts. | Adaptive: adjustment to evolving guidance. |
| Katapally (2023) | Digital dashboards; real-time data; scalable systems. | Technology use; information availability. | Reciprocal: supports evidence-based DM through real-time analytics. | Evidence-based: data-driven decision support. |
| Khalid (2023) | High data volume; need for clear reports. | Evidence clarity; usability. | Reciprocal: aligns with evidence usability emphasis. | Evidence-based: clarity supports action. |
| Guidolin (2022) | Ethical framework (A4R); iterative review. | Ethical framework application. | Reciprocal: aligns with system-level ethical models. | Ethical: structured ethical framework. |
| Skagerström (2023) | Isolation; workload pressure. | Adapting under strain. | Reciprocal: aligns with adaptive coping under stress. | Adaptive: coping under strain. |
| Pettersson (2023) | Organisational barriers; limited knowledge. | Adapting to constraints and limited knowledge. | Reciprocal: aligns with constraint-driven adaptation. | Adaptive: workaround strategies. |
| Bhat (2023) | Rapid evidence products; skilled personnel. | Knowledge sharing supports DM. | Reciprocal: aligns with rapid evidence generation studies. | Evidence-based: rapid synthesis. |
| Bastani (2022) | Use of evidence in crisis. | Evidence informs DM. | Reciprocal: general alignment with evidence-based studies. | Evidence-based: reliance on evidence. |
| Clyne (2023) | Rapid decisions in crises. | Speed essential. | Reciprocal: aligns with rapid response models. | Rapid: time-critical decisions. |
| Romiti (2025) | Decision processes changed over time. | Adaptation across phases. | Reciprocal: temporal adaptation dimension. | Adaptive: phase-based shifts. |
| Phillips (2023) | Decision bottlenecks. | Adaptation needed. | Reciprocal: highlights constraint-based adaptation. | Adaptive: iterative problem-solving. |
| Doran (2025) | Protocols modified. | Flexibility over rigidity. | Reciprocal: consistency with adaptive models. | Adaptive: flexible protocols. |
| Fenton (2021) | Uncertainty and urgency shaping decisions. | Adapting to conditions. | Reciprocal: aligns with crisis-driven adaptation. | Adaptive: volatile context response. |
| Tseng (2005) | Timing, execution, interaction. | Structured DM. | Refutational: contrasts rapid models. | Sound: structured coordination. |
| Thomas (2009) | Ethical concerns; lack of training. | Ethical capability gaps. | Reciprocal: aligns with ethical model limitations. | Ethical: need for structured support. |
| Rimstad (2015) | Decisions within 30 minutes; experience influence. | Rapid decisions; experience bias. | Reciprocal: aligns with rapid models; adds experiential element. | Rapid: experience-informed urgency. |

# Supplementary File 6: Meta-ethnographic analysis of prioritised factors influencing decision-making (DM): translation matrix with reciprocal/refutational notes

| Reference | First-order constructs | Second-order constructs | Translation notes (reciprocal/refutational) | Third-order construct (prioritised factor) |
| --- | --- | --- | --- | --- |
| Edwards (2024) | Adjusting to organisational and staff needs. | Considering organisational and workforce needs. | Reciprocal: aligns with organisational constraint studies; overlaps with urgency under time pressure. | Institutional requirements + urgency: prioritising organisational and workforce needs under time pressure. |
| Johnson (2023) | Managing organisational conflict. | Prioritising conflict resolution. | Reciprocal: aligns with teamwork/coordination factors. | Intra-organisational conflict: prioritising conflict resolution to enable coordination. |
| Johnson-Cornet (2023) | Supporting ethical decision-making at all levels. | Prioritising ethics. | Reciprocal: aligns with ethical studies. | Ethical issues: ethics as a core criterion. |
| Barishansky (2022) | Need for structured decision-making approach. | Prioritising policies and procedures. | Reciprocal: aligns with knowledge; contrasts urgency improvisation. | Knowledge: prioritising structured guidance. |
| Aragón (2021) | Reliance on collective knowledge and intuition. | Prioritising knowledge. | Reciprocal: aligns with knowledge studies; adds intuition. | Knowledge: collective expertise guides high-stakes decisions. |
| Carbone (2023) | Multiple influencing factors. | Factors prioritised simultaneously. | Reciprocal: integrates multiple factors. | Institutional + risk + personal beliefs: balancing influences. |
| Katapally (2023) | Real-time dashboards and big data. | Prioritising reliable data. | Reciprocal: aligns with evidence-based studies. | Knowledge: real-time data supports decisions. |
| Khalid (2023) | Preference for clear evidence summaries. | Prioritising usable evidence. | Reciprocal: aligns with evidence usability. | Knowledge: prioritising credible evidence. |
| Guidolin (2022) | Continuous ethical re-evaluation. | Prioritising ethical principles. | Reciprocal: aligns with ethical studies. | Ethical issues: maintaining ethical alignment. |
| Skagerström (2023) | Supporting staff capacity. | Shared decisions. | Reciprocal: aligns with teamwork. | Teamwork and group structure: shared decision-making. |
| Pettersson (2023) | Organisational constraints. | Considering organisational needs. | Reciprocal: aligns with Edwards. | Institutional requirements: organisational demands shape decisions. |
| Bhat (2023) | Trust in evidence and skilled teams. | Knowledge enhanced by expertise. | Reciprocal: aligns with evidence studies. | Knowledge: trusted evidence improves decisions. |
| Bastani (2022) | Evidence is essential. | Evidence informs decisions. | Reciprocal: baseline alignment. | Knowledge: evidence essential for decisions. |
| Clyne (2023) | Rapid decisions. | Moving quickly. | Reciprocal: aligns with urgency. | Urgency: prioritising speed. |
| Romiti (2025) | Adjusting decisions over time. | Adapting to information. | Reciprocal: aligns with knowledge evolution. | Knowledge: evolving information guides decisions. |
| Phillips (2023) | Decision bottlenecks. | Using knowledge to overcome. | Reciprocal: aligns with constraint studies. | Knowledge: reducing bottlenecks. |
| Doran (2025) | Balancing immediate and long-term needs. | Immediate response needs. | Reciprocal: aligns with urgency. | Urgency: prioritising immediate response. |
| Fenton (2021) | Ethical concerns in interventions. | Avoiding ethical conflict. | Reciprocal: aligns with ethical and risk. | Risk + ethical issues: mitigating harm. |
| Tseng (2005) | Critical timing in crisis. | Moving quickly. | Reciprocal: aligns with urgency. | Urgency: prioritising timing. |
| Thomas (2009) | Moral challenges and lack of training. | Ethical risk avoidance. | Reciprocal: aligns with ethical studies. | Risk + ethical issues: reducing moral harm. |
| Rimstad (2015) | Decisions within 30 minutes; high stakes. | Rapid decisions. | Reciprocal: aligns with urgency + risk. | Urgency + risk: rapid high-stakes decisions. |

# Supplementary File 7: PRISMA 2020 main checklist

| **Topic** | **No.** | **Item** | **Location** |
| --- | --- | --- | --- |
| **TITLE** |  |  |  |
| **Title** | 1 | Identify the report as a systematic review. | Page 1 |
| **ABSTRACT** |  |  |  |
| **Abstract** | 2 | See the PRISMA 2020 for Abstracts checklist | Supplementary File 7 |
| **INTRODUCTION** |  |  |  |
| **Rationale** | 3 | Describe the rationale for the review in the context of existing knowledge. | Page 6 |
| **Objectives** | 4 | Provide an explicit statement of the objective(s) or question(s) the review addresses. | Pages 6,7 |
| **METHODS** |  |  |  |
| **Eligibility criteria** | 5 | Specify the inclusion and exclusion criteria for the review and how studies were grouped for the syntheses. | Pages 7,8 |
| **Information sources** | 6 | Specify all databases, registers, websites, organisations, reference lists and other sources searched or consulted to identify studies. Specify the date when each source was last searched or consulted. | Page 8 |
| **Search strategy** | 7 | Present the full search strategies for all databases, registers and websites, including any filters and limits used. | Supplementary File 2 |
| **Selection process** | 8 | Specify the methods used to decide whether a study met the inclusion criteria of the review, including how many reviewers screened each record and each report retrieved, whether they worked independently, and if applicable, details of automation tools used in the process. | Page 8 |
| **Data collection process** | 9 | Specify the methods used to collect data from reports, including how many reviewers collected data from each report, whether they worked independently, any processes for obtaining or confirming data from study investigators, and if applicable, details of automation tools used in the process. | Pages 8,9 |
| **Data items** | 10a | List and define all outcomes for which data were sought. Specify whether all results that were compatible with each outcome domain in each study were sought (e.g. for all measures, points, analyses), and if not, the methods used to decide which results to collect. | Page 9 |
|  | 10b | List and define all other variables for which data were sought (e.g. participant and intervention characteristics, funding sources). Describe any assumptions made about any missing or unclear information. | Pages 8,9 |
| **Study risk of bias assessment** | 11 | Specify the methods used to assess risk of bias in the included studies, including details of the tool(s) used, how many reviewers assessed each study and whether they worked independently, and if applicable, details of automation tools used in the process. | Pages 8,9 |
| **Effect measures** | 12 | Specify for each outcome the effect measure(s) (e.g. risk ratio, mean difference) used in the synthesis or presentation of results. | Page 9 |
| **Synthesis methods** | 13a | Describe the processes used to decide which studies were eligible for each synthesis (e.g. tabulating the study intervention characteristics and comparing against the planned groups for each synthesis (item 5)). | Page 9 |
|  | 13b | Describe any methods required to prepare the data for presentation or synthesis, such as handling of missing summary statistics, or data conversions. | Figure 2 |
|  | 13c | Describe any methods used to tabulate or visually display results of individual studies and syntheses. | Figure 3 |
|  | 13d | Describe any methods used to synthesise results and provide a rationale for the choice(s). If meta-analysis was performed, describe the model(s), method(s) to identify the presence and extent of statistical heterogeneity, and software package(s) used. | Page 9 |
|  | 13e | Describe any methods used to explore possible causes of heterogeneity among study results (e.g. subgroup analysis, meta-regression). | N/A |
|  | 13f | Describe any sensitivity analyses conducted to assess robustness of the synthesised results. | Page 5 |
| **Reporting bias assessment** | 14 | Describe any methods used to assess risk of bias due to missing results in a synthesis (arising from reporting biases). | Supplementary File 2 |
| **Certainty assessment** | 15 | Describe any methods used to assess certainty (or confidence) in the body of evidence for an outcome. | Page 4 |
| **RESULTS** |  |  |  |
| **Study selection** | 16a | Describe the results of the search and selection process, from the number of records identified in the search to the number of studies included in the review, ideally using a flow diagram. | Page 6 |
|  | 16b | Cite studies that might appear to meet the inclusion criteria, but which were excluded, and explain why they were excluded. | Page 7 |
| **Study characteristics** | 17 | Cite each included study and present its characteristics. | Page 7 |
| **Risk of bias in studies** | 18 | Present assessments of risk of bias for each included study. | Supplementary File 2 |
| **Results of individual studies** | 19 | For all outcomes, present, for each study: (a) summary statistics for each group (where appropriate) and (b) an effect estimates and its precision (e.g. confidence/credible interval), ideally using structured tables or plots. | Tables |
| **Results of syntheses** | 20a | For each synthesis, briefly summarize the characteristics and risk of bias among contributing studies. | Page 8 |
|  | 20b | Present results of all statistical syntheses conducted. If meta-analysis was done, present for each the summary estimate and its precision (e.g. confidence/credible interval) and measures of statistical heterogeneity. If comparing groups, describe the direction of the effect. | N/A |
|  | 20c | Present results of all investigations of possible causes of heterogeneity among study results. | Pages 8,9 |
|  | 20d | Present results of all sensitivity analyses conducted to assess the robustness of the synthesized results. | Page 8 |
| **Reporting biases** | 21 | Present assessments of risk of bias due to missing results (arising from reporting biases) for each synthesis assessed. | Page 7 |
| **Certainty of evidence** | 22 | Present assessments of certainty (or confidence) in the body of evidence for each outcome assessed. | Supplementary File 3 |
| **DISCUSSION** |  |  |  |
| **Discussion** | 23a | Provide a general interpretation of the results in the context of other evidence. | Page 16 |
|  | 23b | Discuss any limitations of the evidence included in the review. | Page 19 |
|  | 23c | Discuss any limitations of the review processes used. | Page 19 |
|  | 23d | Discuss implications of the results for practice, policy, and future research. | Pages 17,18 |
| **OTHER INFORMATION** |  |  |  |
| **Registration and protocol** | 24a | Provide registration information for the review, including register name and registration number, or state that the review was not registered. | Page 7 |
|  | 24b | Indicate where the review protocol can be accessed, or state that a protocol was not prepared. | DOI:10.1155/2024/2038608 |
|  | 24c | Describe and explain any amendments to information provided at registration or in the protocol. | N/A |
| **Support** | 25 | Describe sources of financial or non-financial support for the review, and the role of the funders or sponsors in the review. | UTS university |
| **Competing interests** | 26 | Declare any competing interests of review authors. | Page 1 |
| **Availability of data, code and other materials** | 27 | Report which of the following are publicly available and where they can be found: template data collection forms; data extracted from included studies; data used for all analyses; analytic code; any other materials used in the review. | Page 2 |

*From:* Page MJ, McKenzie JE, Bossuyt PM, Boutron I, Hoffmann TC, Mulrow CD, et al. The PRISMA 2020 statement: an updated guideline for reporting systematic reviews. MetaArXiv. 2020, September 14. DOI: 10.31222/osf.io/v7gm2. For more information, visit: [www.prisma-statement.org](https://studentutsedu-my.sharepoint.com/personal/ehmaidy_m_alqafan_student_uts_edu_au/Documents/Documents/PhD%20folder/UTS%20UNIVERSTY/systemic%20review/www.prisma-statement.org)

# Supplementary File 8: PRISMA 2020 abstract checklist

| **Topic** | **No.** | **Item** | **Reported?** |
| --- | --- | --- | --- |
| **TITLE** |  |  |  |
| **Title** | 1 | Identify the report as a systematic review. | Yes |
| **BACKGROUND** |  |  |  |
| **Objectives** | 2 | Provide an explicit statement of the main objective(s) or question(s) the review addresses. | Yes |
| **METHODS** |  |  |  |
| **Eligibility criteria** | 3 | Specify the inclusion and exclusion criteria for the review. | Yes |
| **Information sources** | 4 | Specify the information sources (e.g. databases, registers) used to identify studies and the date when each was last searched. | Yes |
| **Risk of bias** | 5 | Specify the methods used to assess risk of bias in the included studies. | Yes |
| **Synthesis of results** | 6 | Specify the methods used to present and synthesise results. | Yes |
| **RESULTS** |  |  |  |
| **Included studies** | 7 | Give the total number of included studies and participants and summarise relevant characteristics of studies. | Yes |
| **Synthesis of results** | 8 | Present results for main outcomes, preferably indicating the number of included studies and participants for each. If meta-analysis was done, report the summary estimate and confidence/credible interval. If comparing groups, indicate the direction of the effect (i.e. which group is favored). | Yes |
| **DISCUSSION** |  |  |  |
| **Limitations of evidence** | 9 | Provide a brief summary of the limitations of the evidence included in the review (e.g. study risk of bias, inconsistency and imprecision). | Yes |
| **Interpretation** | 10 | Provide a general interpretation of the results and important implications. | Yes |
| **OTHER** |  |  |  |
| **Funding** | 11 | Specify the primary source of funding for the review. | No |
| **Registration** | 12 | Provide the register name and registration number. | Yes |

*From:* Page MJ, McKenzie JE, Bossuyt PM, Boutron I, Hoffmann TC, Mulrow CD, et al. The PRISMA 2020 statement: an updated guideline for reporting systematic reviews. MetaArXiv. 2020, September 14. DOI: 10.31222/osf.io/v7gm2. For more information, visit: [www.prisma-statement.org](https://studentutsedu-my.sharepoint.com/personal/ehmaidy_m_alqafan_student_uts_edu_au/Documents/Documents/PhD%20folder/UTS%20UNIVERSTY/systemic%20review/www.prisma-statement.org)

# Supplementary File 9: eMERGe checklist

| Phase 1—Selecting meta-ethnography and getting started | | | |
| --- | --- | --- | --- |
| *Introduction* | | | Location |
| 1 | Rationale and context for the meta-ethnography | Describe the gap in research or knowledge to be filled by the meta-ethnography, and the wider context of the meta-ethnography | Page 6,7 |
| 2 | Aim(s) of the meta-ethnography | Describe the meta-ethnography aim(s) | Page 6 |
| 3 | Focus of the meta-ethnography | Describe the meta-ethnography review question(s) (or objectives) | Page 7 |
| 4 | Rationale for using meta-ethnography | Explain why meta-ethnography was considered the most appropriate qualitative synthesis methodology | Page 7 |
| Phase 2—Deciding what is relevant | | | |
| *Methods* | | | |
| 5 | Search strategy | Describe the rationale for the literature search strategy | Page 7,8 |
| 6 | Search processes | Describe how the literature searching was carried out and by whom | Page 8 |
| 7 | Selecting primary studies | Describe the process of study screening and selection, and who was involved | Page 8 |
| *Findings* | | |  |
| 8 | Outcome of study selection | Describe the results of the study searches and screening | Page 9-15 |
| Phase 3—Reading included studies | | | |
| *Methods* | | | |
| 9 | Reading and data extraction approach | Describe the reading and data extraction method and processes | Page 9 |
| *Findings* | | | |
| 10 | Presenting characteristics of included studies | Describe characteristics of the included studies | Page 9,10 |
| Phase 4—Determining how studies are related | | | |
| *Methods* | | | |
| 11 | Process for determining how studies are related | Describe the methods and processes for determining how the included studies are related:  - Which aspects of studies were compared  **AND**  - How the studies were compared | Page 9 |
| *Findings* | | | |
| 12 | Outcome of related studies | Describe how studies relate to each other | Page 9 |
| Phase 5—Translating studies into one another | | | |
| *Methods* | | | |
| 13 | Process of translating studies | Describe the methods of translation**:**  - Describe steps taken to preserve the context and meaning of the relationships between concepts within and across studies- Describe how the reciprocal and refutational translations were conducted- Describe how potential alternative interpretations or explanations were considered in the translations | Page 9 |
| *Findings* | | | |
| 14 | Outcome of translation | Describe the interpretive findings of the translation. | Tables 1,2 |
| Phase 6—Synthesising translations | | | |
| *Methods* | | | |
| 15 | Synthesis process | Describe the methods used to develop overarching concepts (“synthesised translations”)  Describe how potential alternative interpretations or explanations were considered in the synthesis | Page 9  Figure 1 |
| *Findings* | | | |
| 16 | Outcome of the synthesis process | Describe the new theory, conceptual framework, model, configuration or interpretation of data developed from the synthesis | Page 10 |
| Phase 7—Expressing the synthesis | | | |
| *Discussion* | | | |
| 17 | Summary of findings | Summarise the main interpretive findings of the translation and synthesis, and compare them to existing literature | Page 10- 13 |
| 18 | Strengths, limitations, and reflexivity | Reflect on and describe the strengths and limitations of the synthesis:  - Methodological aspects—for example, describe how the synthesis findings were influenced by the nature of the included studies and how the meta-ethnography was conducted.  - Reflexivity—for example, the impact of the research team on the synthesis findings | Page 16-19 |
| 19 | Recommendations and conclusions | Describe the implications of the synthesis | Page 19 |
